# Modular PheWAS reveals the therapeutic heterogeneity landscape of Danghong injection on stable angina pectoris

**DOI:** 10.1186/s43556-026-00517-1

**Published:** 2026-07-18

**Authors:** Bing Li, Jun Liu, Siwei Tian, Lixing Zhu, Dayue Darrel Duan, Zhong Wang

**Affiliations:** 1 Institute of Chinese Materia Medica, China Academy of Chinese Medical Sciences, Beijing, 100700 China; 2 Institute of Basic Research in Clinical Medicine, China Academy of Chinese Medical Sciences, Beijing, 100700 China; 3 Center for Statistics and Data Science, Beijing Normal University at Zhuhai, Guangdong, 519000 China; 4 Department of Mathematics, Hong Kong Baptist University, Hong Kong, China; 5 School of Integrated Medicine, Nanjing University of Chinese Medicine, Nanjing, Jiangsu 210023 China; 6 Department of Pharmacology, University of Nevada Reno School of Medicine, Reno, NV 89557 USA

**Keywords:** Stable angina pectoris, Multi-target therapy, Phenomics, Modular pharmacology, Personalized therapy

## Abstract

Stable angina pectoris (SAP) is a common risk factor for myocardial infarction and death. The genomic, transcriptomic, and phenomic heterogeneities of SAP require combination therapies. Multi-target drugs with fixed doses are emerging as scalable and promising prevention and therapeutic strategies for SAP. However, the identification of targets and underlying mechanisms of multi-target drugs for optimal clinical efficacy remains insurmountable. Here, we report a modular phenome-wide association study (MoPheWAS) of *Danhong* Injection (DHI), a clinically proven effective anti-SAP drug. The interrelated multitargets of DHI were divided into 32 *On-modules*. Eleven clinical SAP phenotypes defined by the Seattle Angina Questionnaire and serum lipids were interactively associated with 14 DHI efficacy-related *Phe-modules.* The intrinsic polymorphic associations between SAP phenotypes and *Phe-modules* manifested systematic positive/negative regulation of specific one-to-one as well as complex many-to-many relationships. These *Phe-modules* were conserved, transformed, or newly emerged modules involving G-protein-coupled receptor signaling and kinase binding. In vitro RNAi analysis confirmed the hub genes GALR1/BCMO1 in Mod-29 as multiple targets for the anti-ischemic effect of DHI. The landscape of modular regulators and polymorphic phenomic associations revealed the multi-target mechanisms underlying the therapeutic heterogeneity of SAP. Therefore, MoPheWAS may provides a novel systematic paradigm for identifying multiple phenomic targets of complex polygenic diseases.

**Supplementary Information:**

The online version contains supplementary material available at 10.1186/s43556-026-00517-1.

## Introduction

The polygenic, transcriptomic, and phenotypic characteristics of complex diseases require multi-targeted therapies to improve the comprehensive efficacy of the multiple clinical phenotypes [1, 2]. As scalable and promising therapy strategies with fixed doses or multi-target drugs are emerging to reduce the prevalence and mortality rates of complex diseases including coronary artery disease (CAD) [3–5]. However, identifying the multiple targets and understanding the therapeutic mechanisms of efficacy-dependent phenotypic heterogeneity of complex disease are challenging.

Stable angina pectoris (SAP) is a common risk factor for myocardial infarction and death [6]. DHI is a herbal multi-target drug extracted from *Radix Salviae miltiorrhizae* and *Flos carthami* that is commonly prescribed for treating SAP [7, 8]. The fingerprint electropherogram obtained through high-performance liquid chromatography confirmed five primary DHI components, including danshensu sodium, protocatechualdehyde, P-coumaric acid, rosmarinic acid, and salvianolic acid B, and the quality control of the similarity among the seven batches reached 99.5% [9]. Studies have shown that the pharmacological mechanisms of DHI are multi-targeted and regulate multiple pathways involving inflammation, oxidative stress, atherosclerosis, coagulation, apoptosis, and hyperlipidemia [9–11]. Despite the reported clinical efficacy of DHI in SAP treatment [12], the multi-target mechanisms underlying therapeutic heterogeneity in different clinical phenotypes remain unclear.

The conventional single-target paradigm has failed to reveal the therapeutic mechanisms of multi-target drugs for complex diseases concerning systems biology and multi-omics networks [13–15]. Genome-wide association studies have identified many single nucleotide polymorphisms (SNPs); however, they do not consider the genetic interactions that determine the heterogeneous phenotypes of diseases [16, 17]. The recently emerged phenome-wide association study (PheWAS), which involves the analysis of various characteristic traits or phenotypes, allows the study of intertwined biological processes that lead to pleiotropic associations with genomic variations. For polygenic and polyphenotypic data comprising binary and continuous variables, PheWAS requires specialized analytical approaches to elucidate the global associations between multitargets and the disease phenome [18]. In biological networks, highly correlated genes usually coordinate as functional clusters, defined as modules that maintain internal environmental homeostasis [19–21]. Therefore, a rewired modular network with functional subunits is more adaptable than an entangled monolithic network [22]. Module-based strategies, rather than single ingredients and targets, are becoming increasingly essential for revealing the relationships between multi-target drugs and the disease phenotype [23, 24]. Studies have elucidated modular mechanisms in complex diseases, such as cancer, and revealed an association between modules and disease phenotypes [25–29]. The gene co-expression relationship manifested as intramodular edge rewiring after treatment, and the drug-responsive allosteric module was identified as the drug’s *On-module* [30]. Rather than a single genetic/phenotypic study, creating a new modular-based research paradigm is essential for understanding the complex genome-phenome relationships for multi-target therapies [18].

In this study, we used a modular phenome-wide association study (MoPheWAS) approach to link multiple assembled targets and clinical phenotypes for SAP therapy based on high-throughput transcriptome-wide sequencing of mRNA data from patients with SAP treated with DHI. Multiple targets were layered into characterized modular regulators (including *On-modules* and *Phe-modules*) to correlate with the efficacy-dependent clinical SAP phenotypes, revealing the landscape of multi-targeted mechanisms of therapeutic heterogeneity for SAP.

## Results

### Identified *On-modules* for SAP therapy

**Fig. 1:**
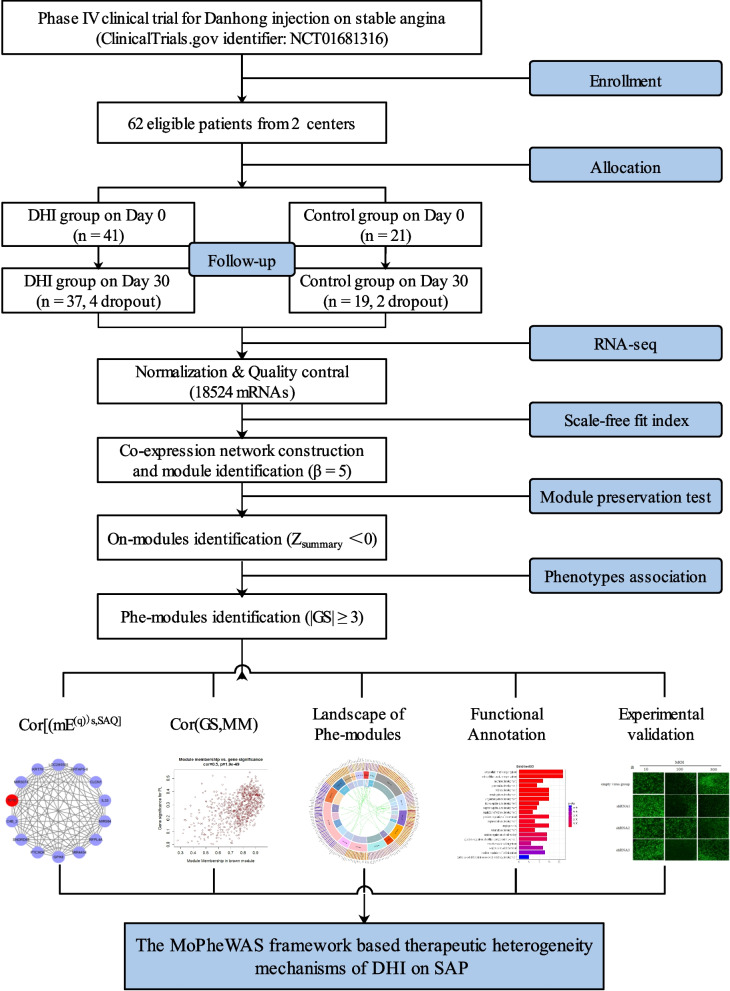
General flowchart of the MoPheWAS framework used in this study. It demonstrates the workflow of dataset processing, network construction, module preservation, *On-modules* identification, *Phe-modules* identification, correlation analysis with clinical phenotypes, and topological and functional analyses

The mRNA expression profiles of 62 patients with SAP in the DHI treatment group on Day 0 (DHD0, *n* = 41) and Day 30 (DHD30, *n* = 37, after excluding 4 dropout), and the placebo control group on Day 0 (CGD0, *n* = 21) and Day 30 (CGD30, *n* = 19, after excluding 2 dropout) were used for co-expression network and MoPheWAS analysis. An overview of the framework used in this study is presented in Fig. 1.

**Fig. 2:**
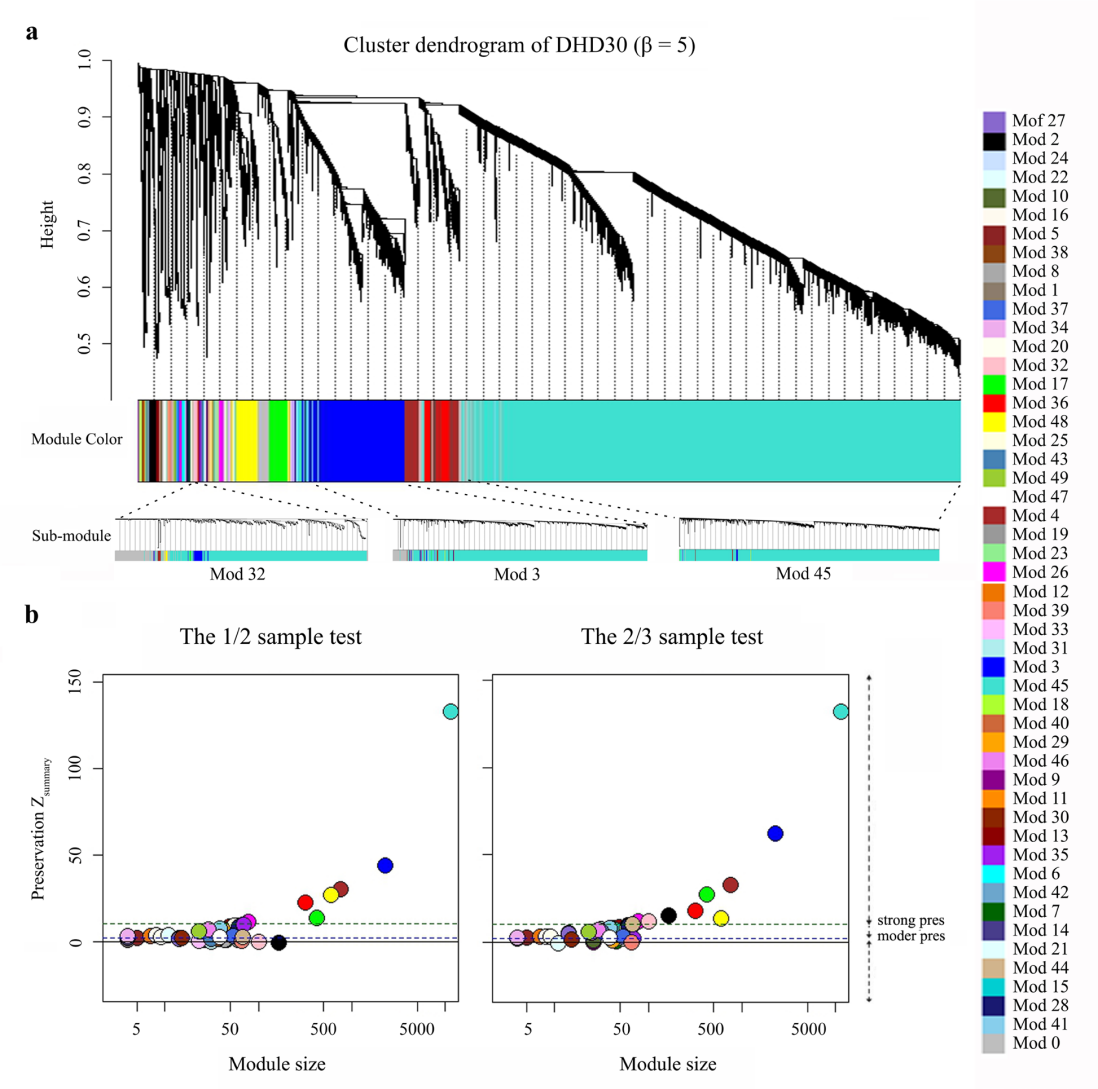
Co-expression modules of DHD30. **a** The hierarchical clustering dendrogram was divided into 49 modules using a dynamic tree-cut algorithm (β = 5). Each cluster tree branch of the dendrogram represents a module, and the various colored bars below the dendrogram denote the corresponding modules. The number of differently colored modules is annotated in the right legend. The sub-modules of *Mod_3, Mod_32,* and *Mod_45* are listed below. **b** A Z_summary_ score statistic was applied to quantify the preservation of each module; 1/2 and 2/3 sample tests are shown in the plots; each colored point represents the corresponding-colored module as in (**a**). Z_summary_ of > 10 indicates strong evidence of preservation, 2 < Z_summary_ < 10 indicates moderate evidence of preservation, whereas Z_summary_ of < 2 indicates no preservation. The green and blue dotted lines indicate the boundary of strong and moderate evidence, respectively

Based on the DHD30 co-expression network of SAP, the hierarchical clustering procedure in weighted gene co-expression analysis (WGCNA) revealed 49 modules (size range: 4–11, 500 mRNAs), and each module was named by a unique color and number in Fig. 2a and Fig. S1, whereas 901 mRNAs labeled by grey and *Mod_0* were not assigned to any module; the specific module composition and its connectivity are presented in Table S1. After the random 2/3 and 1/2 sample splitting preservation tests using Z_summary_ statistics and removing three un-preserved modules (Mod_7, 9, and 39), 46 preserved modules were identified (Fig. 2b; Table S2). Among these 46 modules, eight preserved modules with over 100 genes were re-divided using the same procedure, and 43 sub-modules were obtained (Table S1).

**Fig. 3:**
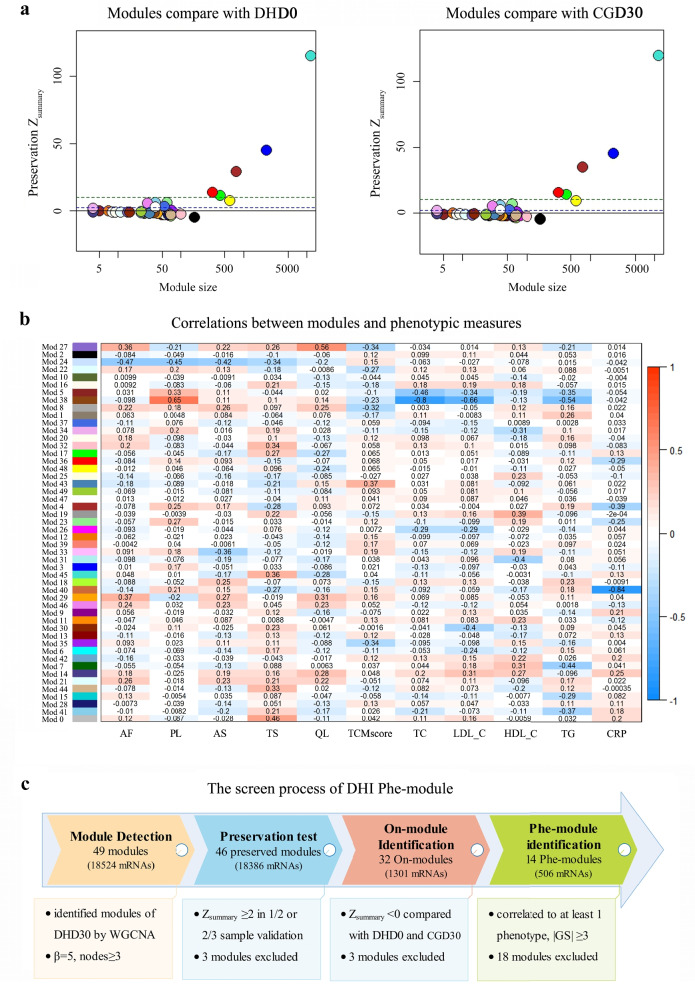
Identification of *On-module*s and their correlations with 11 clinical phenotypes. **a** The Z_summary_ values of the modules were compared between DHD0 and CGD30; modules with a negative Z_summary_ in both comparisons were defined as *On-module*s. The plot legend is the same as that in Fig. 1b. **b** Heatmap showing the correlation coefficients between the modules and clinical phenotypes. Each row corresponds to a module eigengene, and each column corresponds to the phenotype. The table is color-coded using correlation coefficients according to the color legend. **c** Screening process for *On-module* and *Phe-module* for SAP therapy

Compared with the DHD0 and CGD30 of SAP, the preserved modules with a negative Z_summary_ in both comparisons were considered *On-modules*, which may be potential *On-modular* multiple targets for DHI in SAP treatment; 32 *On-modules* with a Z_summary_ of < 0 were identified (size range: 4–163). The detailed Z_summary_ values for all modules are presented in Fig. 3a and Table S2. Among them, seven *On-modules*, including *Mod_2* and *Mod_18*, had a Z_summary_ of < −2 in both comparisons.

### SAP clinical phenotypes were correlated with *Phe-modules*

We assessed whether the *On-modul*es were related to clinical phenotypes in the treatment of the SAP phenome, that is, the *Phe-modules*. The correlation coefficient of each module and *p*-value between their E^(q)^ and variation in 11 phenotypic measures (angina frequency (AF), physical limitation (PL), angina stability (AS), treatment satisfaction (TS), quality of life (QL), Traditional Chinese Medicine blood stasis syndrome scores (TCM score), total cholesterol (TC), low-density lipoprotein-cholesterol (LDL-C), high-density lipoprotein-cholesterol (HDL-C), triglyceride (TG), and C-reactive protein (CRP)) are shown in Fig. 3b, Fig. S2, and Table S3. Of the 32 *On-modules*, 16 *Phe-modules* (including two sub-modules) were associated with at least one clinical phenotype. Through module detection, preservation test, On-module identification and Phe-module identification four steps of dimensionality reduction, 506 mRNAs in these 16 *Phe-modules* were screened as efficacy-related multiple targets for SAP (Fig. 3c), and each *Phe-module* was named based on its functional association with certain clinical phenotypes (Table S2).

**Fig. 4:**
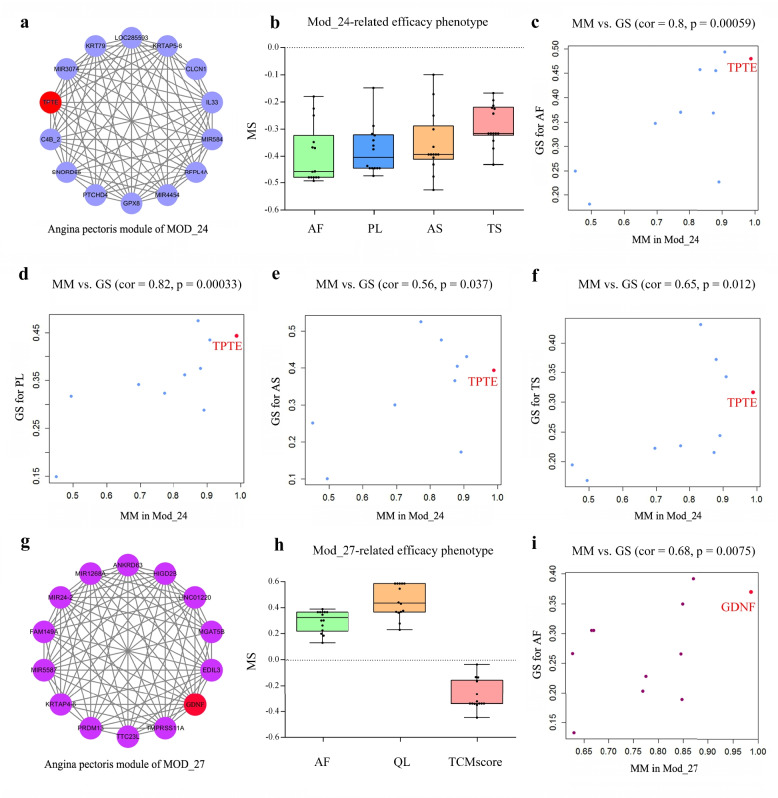
Angina pectoris *Phe-module*-related phenotypes and their hub genes. **a** Visualization of *Mod_24* (a positively regulated module of angina pectoris), a pluripotent module associated with multiple phenotypes. Red nodes highlight the hub genes of TPTE (MM = 0.99), which were also differentially expressed genes (DEGs). **b** Negative correlation between the module significance (MS) of *Mod_24* and angina frequency (AF), physical limitation (PL), anginal stability (AS), and treatment satisfaction (TS) phenotypes. **c-f** Positive correlations between module membership (MM, x-axis) and gene significance (GS, y-axis) of the AF, PL, AS, and TS genes in *Mod_24*. The red points highlight the hub genes of TPTE. **g** Another pluripotent module *Mod_27* (angina pectoris negatively regulated). The red node highlights the hub gene, GDNF (MM = 0.99). **h** Positive correlation between the MS of *Mod_27* and AF and quality of life (QL) phenotypes, and negative correlation with TCM score. **i** Positive correlation between MM (x-axis) and GS (y-axis) of the AF for genes in *Mod_27*. Red points highlight the GDNF hub genes (MM = 0.99)

**Fig. 5:**
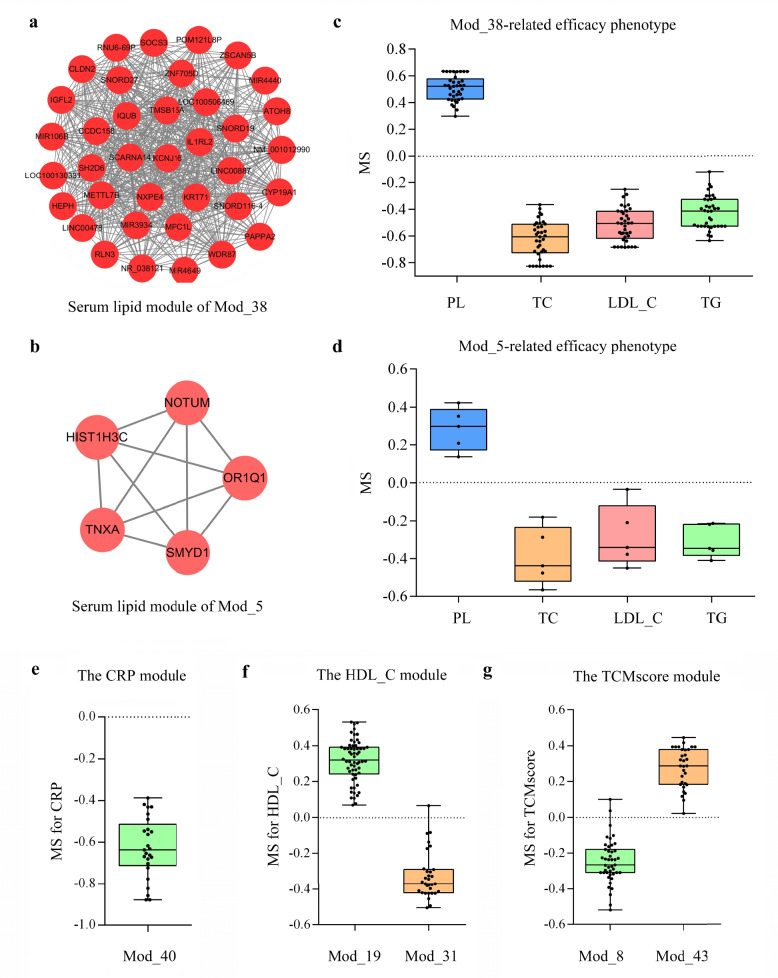
Polymorphic relationships between *Phe-module*s and SAP clinical phenotypes. **a**, **b** Pluripotent blood fat negatively regulates *Mod_38* and *Mod_5*. **c**, **d** This is consistent with the clinical phenotypes of *Mod_38* and *Mod_5*, which were positively correlated with PL levels and negatively correlated with TC, LDL-C, and TG levels. **e** Strong correlation between CRP-specific Mod_40. **f** Two HDL-C-specific modules, *Mod_19* and *Mod_31*, were inversely correlated. **g** Two CMC score-specific modules, *Mod_8* and *Mod_43*, also showed an inverse correlation

While 18 *On-modules* showed no association with the clinical phenotypes, some pluripotent modules were associated with multiple clinical phenotypes (Fig. 3b and Fig. S2). Eight modules (*Mod_5, 24, 27, 29, 32, 33, 38,* and *44*) were associated with at least one specific scale on the Seattle Angina Questionnaire (SAQ). Notably, *Mod_24* (angina pectoris negatively regulated module) was simultaneously associated with four SAQ scales (AF, PL, AS, and TS) (Fig. 4a-f), *and Mod_27* (angina pectoris positively regulated module) was associated with two SAQ scales (AF and QL) and the TCM score (Fig. 4g-i). In addition, the serum lipid-negatively regulated *Mod_5* and *Mod 38* were consistently associated with the four clinical phenotypes (PL, TC, LDL-C, and TG) (Fig. 5a-d). However, some modules were correlated with only one clinical phenotype. For example, the CRP-specific *Mod_40* (Fig. 5e), which had a high correlation coefficient of −0.84.

### Hub genes underlying *Phe-modules* based on modular connectivity

In the gene co-expression network, highly connected hub genes in a module had high gene significance (GS). Similarly, regarding the efficacy-related *Phe-modules*, genes with high module membership (MM) had high GS. The detailed MM values for the *Phe-modules* are listed in Table S4. For the primary clinical phenotype of AF, there’s one case of angina pectoris negatively regulated the *Phe-module* (*Mod_24)*, and two cases positively regulated *Phe-module*s (*Mod_27 and 29*). The GS of *the Mod_24* genes for AF, PL, and TS showed a stronger association with MM; its hub differentially expressed gene (DEG) of TPTE (MM = 0.99) had a high GS (Fig. 4c-f). The GS of *the Mod_27* genes for AF, QL, and TCM scores also had a stronger association with MM, and the hub gene GDNF (MM = 0.99) consistently showed a high GS for the three measures (Fig. 4g-i and Fig. S3a-b). CDH9, TMEM145, SEBOX, BCMO1, and GALR1, with a high GS in AF-specific *Mod_29,* were also hub genes according to MM (Fig. S3c-e). For other clinical phenotypes, the correlations between the GS and MM of *the Phe-modules* are shown in Fig. S3f-o and S4a-o. It can be seen that network connectivity and modular functions are organized by hub genes, and the modular structure derived from gene perturbations is closely associated with phenotypic variations across samples.

### Polymorphic associations between SAP Phenotypes and *Phe-modules*

Owing to the transcriptomic and phenomic heterogeneity of SAP therapy, the efficacy-dependent landscape of associations between SAP phenotypes and *Phe-modules* manifests as systematic positive/negative regulation of specific one-to-one or complex many-to-numerous relationships. According to the positive and negative relationships of phenotypic associations, specific *Phe-modules* can consistently regulate certain clinical phenotypes. For example, the serum lipid modules *Mod_38* and *Mod_5* positively regulated PL and negatively regulated TC, LDL-C, and TG (Fig. 5a-d), and angina pectoris modules *Mod_24* and *Mod_33* can negatively regulate AS (Fig. 4a and Fig. S3g). In contrast, some *Phe-modules* can have opposing regulatory effects on certain clinical phenotypes. Serum lipid modules *Mod_19* and *Mod_31* positively and negatively regulated HDL-C, respectively. The *Phe-modules Mod_8* and *Mod_43* positively and negatively regulated the TCM score, respectively (Fig. 5f-g).

**Fig. 6:**
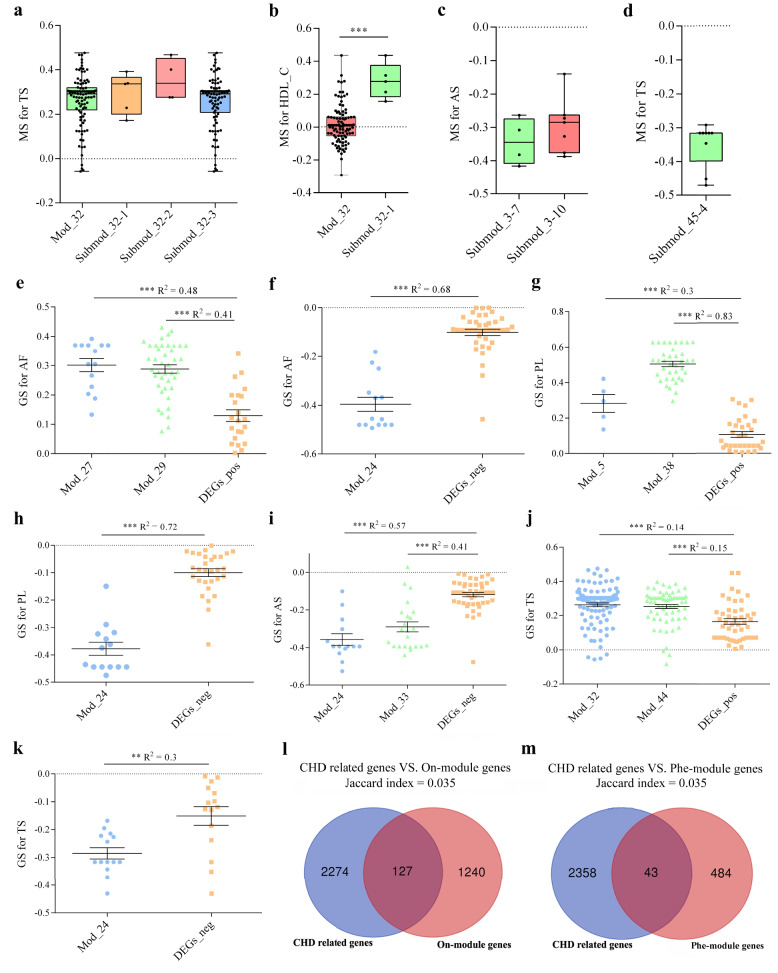
Comparison of *Phe-module*s with sub-modules, DEGs, and CHD-related genes. **a**The sub-modules of angina pectoris positively regulated *Mod_32* and showed consistent correlations with TS. **b** An additional correlation was found between *Submod_32-1* and HDL-C compared with *Mod_32*. **c** Two sub-modules of non-phenotype-related *Mod_3* were correlated with AS. **d** *Submod_45-4* of non-phenotype-related *Mod_45* was correlated with TS. **e-k** For the AF, PL, AS, and TS clinical phenotypes, positively and negatively correlated modules had a higher GS than the positively or negatively correlated DEGs. See also Fig. S5 for additional measures. **l**, **m** Venn diagram demonstrating the number of overlapping genes between CHD phenotype-related genes and *On-module* or *Phe-module* genes. Approximately 10% of the genes in *On-module* or *Phe-module* genes were related to CHD

In addition, the phenotypic correlations of sub-modules were always conserved in the original module, and that the decomposition of sub-modules may help identify the underlying phenotypic correlations. For example, the *Phe-module Mod_32*, which positively regulates T and S, had three sub-modules that were positively correlated with TS, and *Submod_32-1* was also correlated with HDL-C (Figs. 2a and 6a-b). *On-modules Mod_3* and *Mod_45* had no phenotypic correlations; however, their sub-modules were correlated with AS and TS (Figs. 2a and 6c-d).

### Functional assessment and annotation of modular regulators

To assess the functional advantages of these modular regulators, we compared the GS of *the Phe-modules* and DEGs in each clinical phenotype. There were six overlapping genes between *the Phe-modules* and the DEGs of DHD30 (39 upregulated and 23 downregulated) (Table S5). Given the lack of a co-expression relationship, the GS values of positively and negatively correlated DEGs were significantly lower than those of the *Phe-modules* in all clinical phenotypes (Fig. 6e-k and Fig. S5). To demonstrate the functional correlations between the *On*- and *Phe-modules* for SAP, we compared these modular regulators (1,367 and 527 mRNAs in the *On-* and *Phe-modules*, respectively) with 2,401 known CAD-related genes collected from the database and identified 127 and 43 overlapping genes, with Jaccard similarity coefficients of 0.035 and 0.015, respectively (Fig. 6l-m).

Gene Ontology (GO) and Kyoto Encyclopedia of Genes and Genomes (KEGG) pathway enrichment analyses were performed to characterize the biological functions of the *On-modules* and *Phe-modules*. All significantly enriched GO terms and KEGG pathways (*P* < 0.05) are presented in Table S6. The *On-modules* and *Phe-modules* were enriched in functions such as cardiac muscle tissue morphogenesis (GO:0055008), potassium ion transmembrane transport (GO:0071805), cholesterol homeostasis (GO:0042632), cardiac conduction (GO:0061337), GO functions, TGF-beta signaling pathway, and PI3K-Akt signaling pathway. These functions are closely associated with the pathological and therapeutic mechanisms of CAD. Compared with the control treatment (CGD30), the functional annotation clustering of *Phe-modules* specific to DHI involved protein kinase binding, G-protein-coupled receptor (GPCR) signaling, transcription factor activity, and immune response (Table S6).

### Landscape for the therapeutic heterogeneity of SAP, as revealed by MoPheWAS

**Fig. 7:**
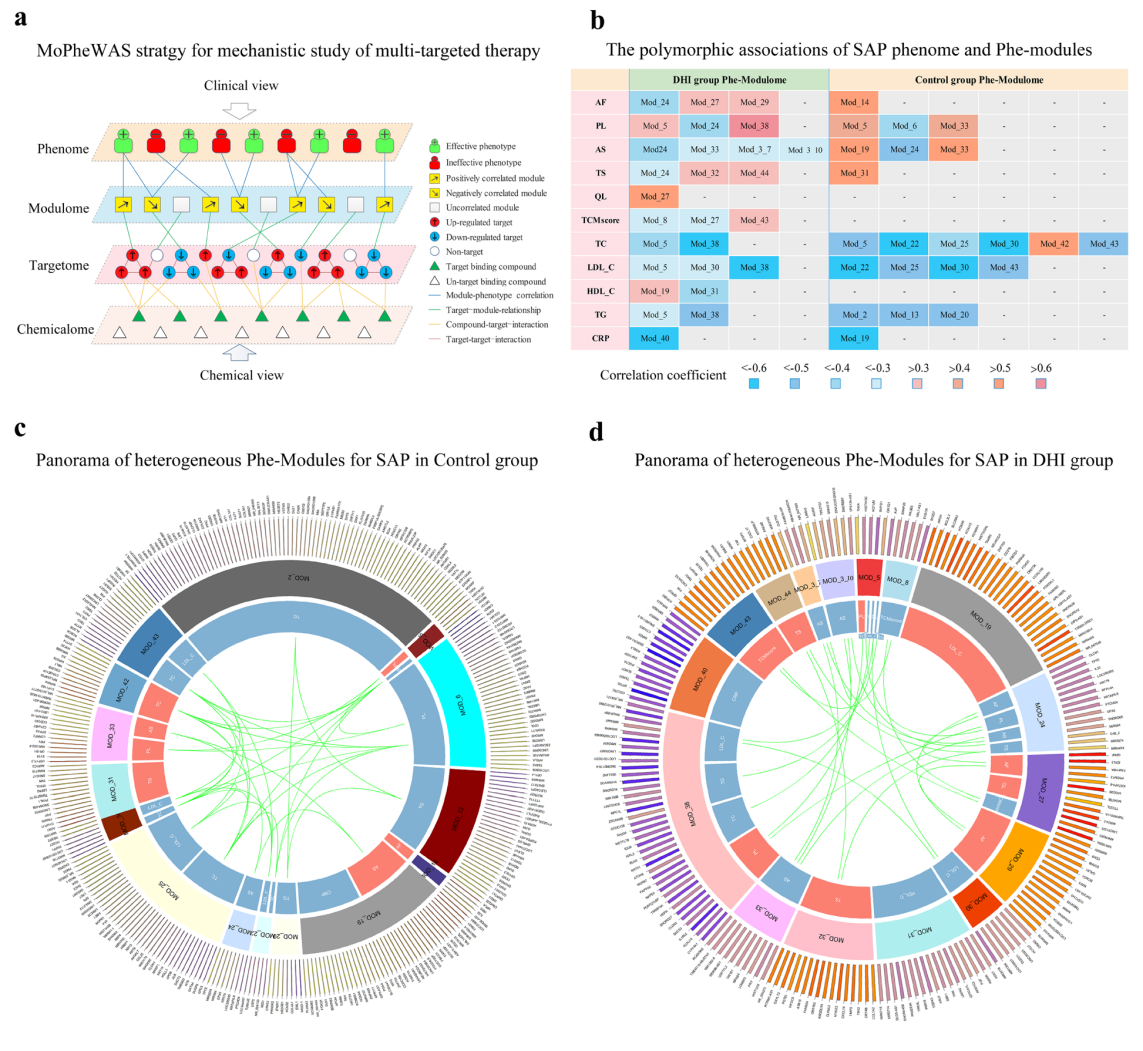
MoPheWAS-based heterogeneous landscape of SAP. **a** Schematic representation of the MoPheWAS strategy for the mechanistic study of multi-target therapy. The legend symbols are labeled at the bottom right. **b** Comparison of SAP clinical phenotype-related *Phe-modules* between the control and DHI groups. **c**, **d** Heterogeneous *Phe-modules* for SAP therapy. The Circos plots of the control (left) and DHI (right) groups contained three concentric tracks from the inside out. The innermost track represents the SAP clinical phenotypes, and different colors correspond to specific *Phe-modules*. The middle track illustrates *the Phe-module*s associated with SAP clinical phenotypes, and the area of a certain module depends on the number of genes it contains. The outer track shows the hub genes of each module, and the color label transition from red to blue indicates a gene’s positive or negative relationship (correlation coefficient from −1 to 1) with a certain phenotype. Green lines in the center linking the same phenotype show the relationships between *Phe-modules* for SAP treatment

By systematically integrating and linking drugs, targets, modules, and clinical phenomes of disease therapy networks, the proposed MoPheWAS paradigm revealed the landscape of phenotypic heterogeneity and multi-targeted therapeutic mechanisms for complex diseases (Fig. 7a). By mapping the *Phe-modules* to the transcriptome data of patients with CGD30, we obtained their clinical phenotypic associations for the control treatment, and the *Phe-modules* of DHI were conserved (preservation of module-phenotype association across groups), transformed (the modules associated with the same phenotype are transferred from one to another), or newly emerged (new associated modules have emerged for a certain phenotype) compared with those of CGD30 (Fig. 7b-d). For example, the *Phe-module Mod_5* was conserved, positively regulating PL and negatively regulating TC. The positively regulated Mod-14 in the control group transformed into three *Phe-modules* (negatively regulated Mod-24 and positively regulated Mod-27 and Mod-29) after treatment, which reduced the AF. *Mod_19* was transformed into *Mod_40* after treatment, which negatively regulated CRP. *Mod_8* was newly emerged to negatively regulated TCM score.

Regarding the landscape of SAP therapy, 16 *Phe-modules* (including two sub-modules) were associated with 11 clinical phenotypes in a polymorphic manner (Fig. 7d). Four pluripotent modules were associated with multiple phenotypes, and 12 modules were linked to only one specific phenotype. Crosstalk among phenotype-related *Phe-modules* manifested as systematic positive/negative relationships. The panorama of SAP clinical phenotypes linking DHI *Phe-modules* and their hub genes with high GS and MM reflected their multi-targeted therapeutic mechanisms, and the same *Phe-modules* also linked similar or opposing clinical phenotypes based on their polymorphic associations (Fig. 7).

### Silencing the hub genes of the AF *Phe-module* changed the anti-ischemic effect

**Fig. 8:**
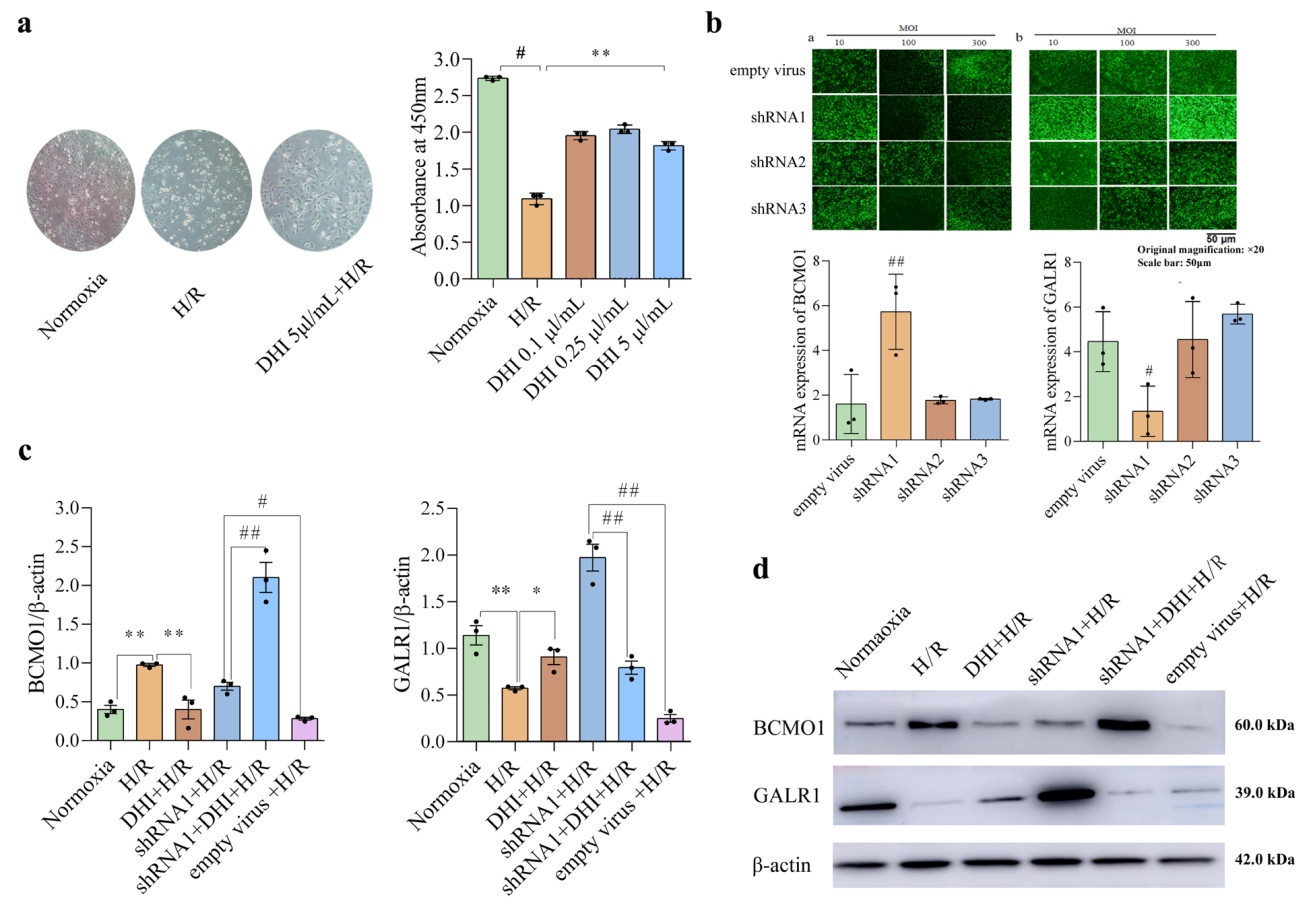
Changes in hub protein expression in the AF *Phe-module* intervened by DHI. **a** Growth status of AC16 cells under normoxia, H/R, and DHI + H/R treatments. (#*P* < 0.05 vs normoxia; **P < 0.01 vs H/R). **b** Changes in fluorescence and mRNA expression of BCO1 and GALR1 in AC16 cells 48 h after intervention with adenoviruses at different MOI. (#*P* < 0.05 vs. empty virus, ##*P* < 0.01 vs. empty virus). **c** Quantification of BCMO1 and GALR1 protein expression levels in each group (Normoxia, H/R, H/R + DHI, shRNA1 + H/R, shRNA1 + DHI + H/R, and empty virus + H/R). (**P < 0.01 vs H/R, ##*P* < 0.01 vs shRNA1 + H/R). **d** Immunoblotting of BCMO1 and GALR1 in each group

The CCK-8 assay revealed that the optical density (OD) in the normoxia group was significantly higher than that in the hypoxia-reoxygenation (H/R) group (*P* < 0.05). After DHI intervention, the OD value in the H/R group was significantly higher than that in the H/R group (*P* < 0.01) (Fig. 8a). According to the cell growth state, 5 μL/mL DHI was used in subsequent experiments. After 48 h of intervention with different multiplicities of infection (MOI) adenoviral solutions, we selected an optimal MOI of 100 according to fluorescence intensity (Fig. 8b). Based on BCMO1 and GALR1 mRNA expression levels (Fig. 8b), the shRNA1 transfection fragment was selected for subsequent western blotting. The protein expression levels of BCMO1 and GALR1 in each group are shown in Fig. 8c-d. The protein expression was BCMO1 in the H/R group than in the normoxia group (*P* < 0.01). The H/R group had significantly higher protein expression of BCMO1 than the DHI + H/R group (*P* < 0.01). The empty virus + H/R group had lower protein expression of BCMO1 than the shRNA1 + H/R and shRNA1 + DHI + H/R groups (*P* < 0.05). GALR1 protein expression was lower in the H/R group than in the normoxia group (*P* < 0.01). The protein expression of GALR1 in the DHI + H/R group was significantly higher than that in the H/R group (*P* < 0.05). Compared with that in the empty virus + H/R group, the protein expression of GALR1 in the shRNA1 + H/R group was significantly increased (*P* < 0.05) and significantly decreased in the shRNA1 + DHI + HR group compared with that in the shRNA1 + H/R group (*P* < 0.01).

## Discussion

The polygenic characteristics of complex diseases, such as SAP, require multi-target therapies, such as herbal drugs. Single-target research strategies have failed to elucidate comprehensive efficacy results owing to the combined influence of multiple variants and heterogeneous phenotypes. Modular rewiring, rather than a single gene, can better reflect integrated responses to multi-target drugs [31–33]. It is essential to depict multitarget-dependent efficacy and phenotypic associations from a modular perspective [23, 34]. Unlike traditional PheWAS studies, we have extended the SNP/gene-based analysis of phenotypic associations to multi-gene modules, and expanded the genetic phenotypes to clinical-based phenome [18]. The proposed MoPheWAS framework aids in deciphering the therapeutic heterogeneity of complex diseases by proportioning multiple targets into phenotype-specific functional modules derived from transcriptome signatures with and without treatment (Fig. 1). Fourteen *Phe-modules* related to 11 SAP clinical phenotypes were assembled into an efficacy-dependent panorama of SAP therapy with DHI. Such studies provide a promising template for identifying phenomic-specific multiple targets to achieve precision medicine for other complex diseases.

Our previous clinical study indicated that 14-day usage of DHI could significantly reduce the number of angina episodes and improve angina-specific health status for at least 90 days, which confirmed the additional benefit and safety of DHI when added to standard medications in patients with SAP [12]. The identified *Phe-modules* manifested polymorphic regulatory characteristics for the SAP-related clinical effects of DHI in SAP treatment phenotypes. A phenotype is usually regulated by multiple modules simultaneously, such as the AF-related *Mod_24*, *Mod_27*, and *Mod_29*, and a *Phe-module* can also regulate multiple clinical phenotypes, such as *Mod_5* and *Mod_28* for PL, TC, LDL-C, and TG levels. However, there were *Phe-modules* that positively or negatively regulated the same phenotypes, such as the HDL-C-specific *Mod_19* and *Mod_31*, allowing us to predict synergistic or antagonistic relationships between *Phe-modules*. *Phe-modules* can also reflect the relationships between diseases and clinical phenotypes. For example, the *Phe-modules* related to CHD risk measures of TC, TG, and LDL-C had a consistent effect direction; however, they were not related to HDL-C, which is inversely associated with CHD [35]. This revealed the clinical efficacy mechanisms of DHI, which can effectively decrease the plasma levels of TC, TG, and LDL-C and increase the levels of HDL-C [36].

In a gene co-expression network, hub genes with high connectivity usually have a high correlation with a trait or clinical phenotype [37]. The use of module eigengene profiles as representative phenotype-related measures has been validated in several studies [26, 38]. In our study, several MM-based hub genes were closely associated with CHD. Taking the AF-specific *Mod_29* as an example (Fig. S3e), the experimentally validated hub gene GALR1 elicits biological effects by interacting with specific GPCRs, which are reportedly rational therapeutic targets for cardiovascular diseases [39]. The other hub gene, BCMO1, affects various metabolic pathways, including those involved in atherosclerosis, and the hub gene ESRRB is critical for regulating the differentiation of embryonic stem cells into cardiovascular lineages [40, 41]. The hub gene TSPAN7 and ANGPTL2 in *Mod_32* are important markers for angiogenesis angiogenesis, which is critical for SAP treatment [42, 43].

DEGs have been conventionally used as markers of drug-responsive targets of disease therapy and can reveal the genetic causes of polygenic diseases, including cardiovascular diseases, in the pre-genome or post-genome eras; however, they have largely proven unsuccessful [44]. A previous study reported that transcriptional profiling-based modular network rewiring analysis was used to predict dynamic drug sensitivity and revealed differentially expressed modules responsible for drug responses that were not shown by DEGs [33]. In our study, the correlations between *Phe-modules* and all phenotypic measures had a significantly higher GS than that of DEGs. These results support the biological significance of the identified *Phe-modules*, possibly enabling the discovery of more response-specific multiple targets at the multi-omics network level.

This study had a few limitations. The clinical phenotypes of SAP are limited, and the sample size was moderate. Besides, the module‑phenotype correlation tests may have potential over-fitting, and additional genes from more Phe-modules may need further experimental and external validations in future Research. Nevertheless, the MoPheWAS framework successfully revealed the efficacy-dependent multi-target and heterogeneous phenotypic association mechanisms of DHI in SAP therapy.

## Conclusion

The present MoPheWAS effectively revealed 32 *On-module* multitargets and 14 *Phe-modules* systematically related to the 11 SAP clinical phenotypes. *Phe-modules* of *Mod_24, Mod_27, Mod_29, Mod_32* and hub genes of GALR1, BCMO1, TSPAN, ANGPTL2 are involving in GPCR signaling, protein kinase binding and angiogenesis. The landscape of modular regulators and polymorphic phenotypic associations revealed the multi-targeted mechanisms underlying therapeutic heterogeneity in SAP. Therefore, MoPheWAS may provides a novel systematic paradigm for identifying multiple phenomic targets in other diseases.

## Materials and methods

### Patients and genomic datasets

Participants aged 42–70 years were recruited for this randomized, double-blind, placebo-controlled clinical trial (ClinicalTrials.gov: NCT01681316) [12, 45], total of 918 patients were randomly assigned to the DHI treatment (40 mL of DHI daily with standard medical care) or placebo (40 mL of 0.9% saline with standard medical care) group at a 2:1 ratio. The diagnostic criteria for SAP were determined according to the Chinese Guidelines for the Diagnosis and Treatment of Chronic Stable Angina (2007) [46], the ACC/AHA Guideline Update for the Management of Patients With Chronic Stable Angina (2002) [47], and the European Society of Cardiology Guidelines for the Management of Stable Angina Pectoris [48]. This trial was approved by the Ethics Committee of the Institute of Basic Clinical Research, China Academy of Chinese Medical Sciences (NO.2013–10), and the Ethics Committee of the Chinese PLA General Hospital (NO.2012–025).

Sixty-two patients with SAP from two centers (41 from the DHI group and 21 from the control group, the baseline information was shown in Table S7) agreed to provide serum samples for RNA sequencing at Day 0 (D0) and Day 30 (D30). The mRNA profiles were obtained using high-throughput sequencing by Illumina HiSeq2000 platform according to the manufacturer’s instructions. The raw data was evaluated by filtering contaminants and initial judgment. The sequencing reads which containing low-quality, adaptor-polluted and high content of unknown base (N) reads were removed. Sequences were aligned to the human genome reference (hg19) sequence, Rfam, and the NCBI database using Bowtie2 [49] (one mismatch allowed) with default parameters. The datasets were deposited in the CNSA (https://db.cngb.org/cnsa/) of the China National GeneBank Database with the accession number CNP0000461. DEGs between D30 and D0 were screened using DESeq2 [50]. Student’s t-test was used to analyze DEGs with a *P* < 0.05 and at least a 1.5-fold change. The gene expression datasets containing 18,524 mRNAs (after removing 2,205 mRNAs with more than half of the values missing) of the DHI treatment group on D0 (DHD0, *n* = 41), D30 (DHD30, *n* = 37, after excluding 4 dropout) and the placebo control group on Day 0 (CGD0, *n* = 21), D30 (CGD30, *n* = 19, after excluding 2 dropout) were selected and used for co-expression network and MoPheWAS analysis (Table S8). An overview of the framework used in this study is shown in Fig. 1.

The clinical study showed that the SAQ scores, including the specific scales of AF, PL, AS, TS, QL, and TCM blood stasis syndrome scores (TCM score) of DHD30, were significantly improved compared with those of CGD30 [12]. Besides the SAQ and TCM scores, the parameters used for the diagnosis and therapeutic efficacy evaluation of SAP, including TC, LDL-C, HDL-C, TG, and high-sensitivity CRP, were selected as SAP phenotypes related to DHI therapy. The clinical phenotypes and their variations are listed in Table S9.

### Discovery of the co-expression module for SAP therapy

We used the WGCNA R package [51] to detect the co-expression modules of SAP based on the transcriptomic dataset of DHD30. As a scale-free distribution property of biological networks, the parameters used in WGCNA to construct the gene co-expression network were based on the topological scale-free criteria. A matrix of pairwise correlations was constructed between all pairs of genes across the measured samples using the appropriate soft-threshold β, which was selected when the network had the best scale-free topology criterion [52]. Here, we chose β = 5 (R^2^ > 0.6, mean connectivity < 1000) for DHD30 co-expression network construction (Fig. S1). The topological overlap measure and Dynamic Hybrid Tree-Cut algorithm were used to perform average linkage hierarchical clustering and to partition the branches of the dendrogram into a module [53], that is, a group of closely related genes. In this study, the module size was set to three.

To assess the preservation of the obtained modules, 2/3 and 1/2 of the samples in the DHD30 were randomly selected as independent test sets, and summary Z-score statistics (Z_summary_) were applied to quantify the preservation of a module topology [54]. The Z_summary_ thresholds were set as follows: Z_summary_ < 2, no preservation; 2 < Z_summary_ < 10, moderate evidence of preservation; and Z_summary_ > 10, strong evidence of preservation [54]. In this analysis, modules with a Z_summary_ of < 2 in the 2/3 and 1/2 sample tests were excluded.

$$Z_{\mathrm{summary}}=\frac{\mathrm{median}({\mathrm Z}_{\mathrm{meanCor}},_{\mathrm{ZmeanAdj}},{\mathrm Z}_{\mathrm{propvarExpl}},{\mathrm Z}_{\mathrm{meamKME}})+\mathrm{median}({\mathrm Z}_{\mathrm{cor}.\mathrm{KIM}},{\mathrm Z}_{\mathrm{cor}.\mathrm{KME}},{\mathrm Z}_{\mathrm{cor}.\mathrm{cor}})}2$$

The significance of the modules detected in DHD30 was assessed by comparing the actual network to potential networks derived from 1,000 permutation tests through randomization of all gene data across samples. For each permutation, the co-expression algorithm was repeatedly run to create modules with the same parameters used for the true network, that is, β = 5, mini-module size = 3. Based on permutation tests, no modules in the actual network could be explained by random chance (*P* < 0.05; Fig. S1b). Due to the numerous genes in DHD30, we further tested whether large, preserved modules (> 100 nodes) could be detected and re-divided into sub-modules to assess the clustering quality of the modules.

### Defining the On-modules for SAP therapy

The Z_summary_ was adopted to quantitatively identify the On-module of DHI in the SAP treatment, which can reveal whether the density and connectivity patterns of modules defined in a reference dataset are preserved in a test dataset [55]. A negative Z_summary_ indicates module disruption [56]; therefore, a Z_summary_ of < 0 was selected as the *On-module* threshold. To obtain a pure drug response to SAP therapy, the DHD30 module was compared with those of DHD0 and CGD30. Modules with a Z_summary_ of < 0 in both comparisons were defined as *On-modules*, which may be potential multi-targeted modules for SAP treatment.

### Identification of Phe-modules for SAP Phenome and its heterogeneity

To further identify *On-modules* that are significantly associated with the therapeutic efficacy of SAP treatment, the module GS to phenotypic measures was computed as the value of the correlation between the *i*-th node profile *x*_*i*_ and the outcome indicator *T.* The higher the GS*i* absolute value, defined as *GSi* =|*cor(x*_*i*_*,T)*|. the more biologically significant the *i*-th gene [57].

Based on GS, the module eigengene [E^(q)^, where q denotes a module] was correlated with clinical efficacy phenotypes, such as the SAQ score, TCM score, and serum lipid levels, which were the differences in outcome measures before and after treatment. E^(q)^ is defined as the first principal component of a given module and is considered to be representative of the gene expression profiles of the module. The correlation coefficient, referred to as the eigengene significance, was computed, and the *p*-value was obtained from a univariate regression model between E^(q)^ and the clinical efficacy phenotypes. For a certain phenotype, modules with a correlation coefficient of > 0.3 and *P* < 0.05 were regarded as positive-regulation *Phe-modules*, and those with a correlation coefficient of < −0.3 and *P* < 0.05 were considered negative-regulation *Phe-modules*. These are efficacy-related modules for the SAP therapeutic phenome.

### Module topological correlations and hub gene identification

Besides eigengene significance, the module significance (MS) was determined as the average GS of all genes in a given module. MS values were highly correlated with eigengene significance [57]. The higher the MS in a module, the more significant the relationship between the module and the clinical phenotypes of interest.

In the co-expression network, the connectivity of a gene is the sum of all adjacencies with all other genes, and intra-module connectivity (K_im_) is the same concept; however, it is only for genes inside a module. K_im_ reveals the degree or co-expressed connectivity of a module. This is a significant variable for screening biologically crucial hub genes [58]. The fuzzy eigengene-based connectivity measure of MM, also known as K_ME_, was used to screen hub genes and explore the relationships between the topological structure and phenotypic significance of a module. The MM measure was significantly related to the K_im,_ and highly connected hub genes had high GS and MM values in specific modules.

### Functional analysis of *On-modules* and *Phe-modules*

To assess the function of a module, the positively and negatively correlated DEGs of DHD30 were considered as presumed ‘module’, and their significance in clinical efficacy phenome correlations was compared with the actual positive- and negative-regulation *Phe-modules*. In addition, 2,401 known CHD-related phenotypic genes were collected from the Human Phenotype Ontology (HPO, https://hpo.jax.org/) and dbGaP (https://dbgap.ncbi.nlm.nih.gov/) databases, which served as reference gene sets for comparison with the *On-modules* and *Phe-modules*. The Jaccard similarity coefficient was used to measure functional similarity.

To further characterize the functions of *On-modules* and *Phe-modules*, GO and KEGG pathway enrichment analyses were performed using the Database for Annotation, Visualization, and Integrated Discovery [59]. All genes in a specific module were uploaded to this database, and the over-representation of a term was defined as a modified Fisher’s exact *p*-value with adjustment for multiple tests using the Benjamini method. With “Homo sapiens” as species background, GO terms and pathways with *P* < 0.05 were considered to be statistically significant.

### *In vitro* RNAi analysis of the Hub genes in AF Phe-modules

To explore the intervention effect of DHI on the *Phe-module*s, an H/R model of AC16 cells was established, and the hub genes BCMO1 and GALR1 in the AF-specific *Phe-module* of MOD_29 were selected for RNA silencing through adenoviral infection. Protein expression of wild-type and RNAi gene fragments with DHI intervention was observed.

### Cell culture and Hr model construction

DHI (Heze, Shandong, China; Lot No. 20081023) was purchased from Shandong Danhong Pharmaceutical Co., Ltd. AC16 cells were purchased from ProCell (Millipore, BH-C304) and cultured in Dulbecco's Modified Eagle Medium (Biosharp, Lot No.: 22109243) supplemented with 10% fetal bovine serum (ExCell Bio, Shanghai, China; catalog No.: FSP500) in an incubator (MCO-18, SANYO) at 37 ℃ and 5% CO_2_. AC16 cells with good growth status were seeded at 5 × 10^3^ cells/well in 96-well plates and divided into five groups: normoxia (cultured in an incubator at 37 ℃ and 5% CO_2_ for 24 h), H/R (after normoxia treatment 24 h, replaced with a low-glucose serum-free medium and placed in 3% O_2_, 5% CO_2_ hypoxic incubator (MCO-15AC, SANYO), and cultured for 24 h), H/R + DHI (H/R + different doses of 0.1, 0.25, and 5 μL/mL DHI). After overnight incubation, 10 μL of CCK-8 (DOJINDO, Lot No.: GX735) was added to each well, and the culture was continued for 2 h at 37 ℃. The absorbance of each well was measured using a microplate reader at an OD of 450 nm (Planck New Technology Co., Ltd., DNM-9602).

### PCR analysis

To identify the optimal MOI and viral fragments, we calculated the mRNA expression of hub genes using quantitative polymerase chain reaction (qPCR). Total RNA was extracted from 96-well-cultured cells using a total RNA assay kit (TIANGEN, Beijing, China; DP419), following the manufacturer’s instructions. A spectrophotometric method was used to measure concentration and integrity of RNA. First, the instrument was set to RNA sample mode, the detection port was cleaned with sterile RNase-free water, and a blank calibration was conducted. Then, 1 μL of RNA sample was loaded onto the detection port, and the RNA concentration, along with the A₂₆₀/A₂₈₀ and A₂₆₀/A₂₃₀ ratios, was recorded. We used the criteria of an A₂₆₀/A₂₈₀ ratio between 1.5–1.9 and an A₂₆₀/A₂₃₀ ratio between 2.0–2.5 to select qualified samples, which helped exclude contamination by salts or organic substances and RNA degradation. RNA integrity was evaluated via denaturing agarose gel electrophoresis. A small amount of RNA sample was denatured, subjected to gel electrophoresis, and stained with ethidium bromide. The integrity was determined by observing the ratio of 28S to 18S rRNA bands: clear bands with a ratio close to 2:1 indicated good RNA integrity, while blurred bands or a deviated ratio suggested partial RNA degradation. EasyScript First-Strand cDNA Synthesis SuperMix (Aibosen Biotechnology Co., Ltd., Beijing, China, PR5801) and SYBR qPCR (Aibosen Biotechnology Co., Ltd., Beijing, China, PR3302) were used for qPCR analysis. We set the PCR thermal cycling conditions to 95 °C for 2 min at pre-denaturation stage, and 95 °C for 5 s → 50–60 °C for 10 s → 72 °C for 15 s of 40 cycles at PCR amplification stage. We set a melting curve formation of 60–95 °C to ensure single product amplification. β-actin was used as an endogenous control, and relative gene expression was calculated using the 2^−ΔΔCT^ method. The primer sequences used for qRT-PCR analysis are listed in Table S10.

### Viral transfection

Adenovirus BCMO1 and GALR1 shRNA-EGFP (Hanheng, AD2209) were purchased from Shanghai Hanheng Bio Company. Adenovirus was melted on ice, and virus solutions with different MOI values were prepared using serum-free DMEM (Biosharp, Lot No.: 22109243) according to the adenovirus operator manual and the virus titer. After 48 h of infection, a fluorescence microscope (OLYMPUS Company, Japan, catalog No.: BX61VS) was used to select the best fragment for further experiments based on the MOI.

### Western blotting

Proteins were extracted from the cell lysates using radioimmunoprecipitation assay (RIPA) buffer (Sigma, Lot No.: QJ222447), and the protein concentration was quantified using a Bicinchoninic Acid Protein Assay Kit (Solarbio, Lot No.: 20221009). Equal amounts of total protein were separated by sodium dodecyl-sulfate–polyacrylamide gel electrophoresis (SDS-PAGE, Epizyme Biomedical Technology Co., Lot No.: 035A8300) and transferred onto a polyvinylidene fluoride (PVDF) membrane (Millipore, Lot No.R1JB27545). The membrane was incubated in 5% skim dry milk (Bio-Ruler, Lot. r1028021) dissolved in Tris-Buffered Saline and Tween 20 (TBST) (Solarbio, Lot No. 20150126) for 2 h. Subsequently, it was incubated with β-actin (Affinity, Rabbit RecAb; Lot No.: 23005014, dilution 1:20,000), BCMO1 (Anti-BCO1 rabbit monoclonal antibody; Lot No.: 335E7A11; dilution: 1:1000), and GALR1 (Anti-GALR1 rabbit monoclonal antibody; Lot No.: CR303368-7, dilution 1:1000) primary antibodies at 4 ℃ overnight, followed by incubation with a horseradish peroxidase (HRP)-conjugated secondary antibody (ProteintechGroup, Inc, Lot No.: GR3355136-10) for 2 h. Immobilon Western A chemiluminescent HRP substrate (Millipore, WBKLS0100) was added dropwise to the PVDF film and incubated until the fluorescent bands became apparent.

### Statistical analysis

All data processing and statistical analyses were performed by R 4.4.1. Correlations were assessed with Pearson’s method. Quantitative data were compared by Analysis of Variance (ANOVA) and t test. Two-sample t-test were employed for comparison between groups. Multiple testing was corrected by the FDR procedure. *P* < 0.05 was considered statistically significant.

## Supplementary Information

Supplementary Material 1.

Supplementary Material 2.

Supplementary Material 3.

Supplementary Material 4.

Supplementary Material 5.

Supplementary Material 6.

Supplementary Material 7.

Supplementary Material 8.

## Data Availability

All data are available in the main text or the supplementary materials. The raw RNA-seq data were deposited in the China National GeneBank Database (accession number: CNP0000461).

## Abbreviations

AF: Angina-frequency scale
AS: Angina stability
CAD: Coronary artery disease
CGD30: Placebo control group on day 30
CRP: C-reactive protein
DEGs: Differentially expressed genes
DHD0: DHI treatment group on day 0
DHD30: DHI treatment group on day 30
DHI: Danhong Injection
GS: Gene significance
GWAS: Genome-wide association study
HDL-C: High-density lipoprotein-cholesterol
LDL-C: Low-density lipoprotein-cholesterol
MM: Module membership
MoPheWAS: Modular phenome-wide association study
On-module: Drug-responsive module compared with control
Phe-module: On-module that related to certain clinical phenotypes
PheWAS: Phenome-wide association studies
PL: Physical limitations
QL: Quality of life
SAP: Stable angina pectoris
SAQ: Seattle Angina Questionnaire
TC: Total cholesterol
TCM score: Traditional Chinese Medicine blood stasis syndrome scores
TG: Triglyceride
TS: Treatment satisfaction
Z_summary_: Summary Z-score statistics for quantify module preservation
