# Supplementary material for: Modular PheWAS reveals the therapeutic heterogeneity landscape of Danghong injection on stable angina pectoris
Source: Mol Biomed. 2026 Jul 18;7:115. doi: 10.1186/s43556-026-00517-1 (PMC13379564; doi:10.1186/s43556-026-00517-1)
Supplement: Supplementary file 1 — Supplementary Material 1. [file 43556_2026_517_MOESM1_ESM.pdf]

Modular PheWAS Reveals the Therapeutic Heterogeneity Landscape of Danghong Injection on Stable Angina Pectoris

Bing Li[1], Jun Liu[2], Siwei Tian[1], Lixing Zhu[3,4], Dayue Darrel Duan[5,6*], Zhong Wang[2*]

[1.]Institute of Chinese Materia Medica, China Academy of Chinese Medical Sciences, Beijing 100700, China

[2.]Institute of Basic Research in Clinical Medicine, China Academy of Chinese Medical Sciences, Beijing 100700, China

[3.]Center for Statistics and Data Science, Beijing Normal University at Zhuhai, Guangdong, 519000, China

[4.]Department of Mathematics, Hong Kong Baptist University, Hong Kong, China

[5.]School of Integrated Medicine, Nanjing University of Chinese Medicine, Nanjing, Jiangsu, 210023, China

[6.]Department of Pharmacology, University of Nevada Reno School of Medicine, Reno, NV, 89557, USA

***Corresponding author**: Dayue Darrel Duan, dduan@medicine.nevada.edu; Zhong Wang, zhonw@vip.sina.com

1. Supplementary Figure 1

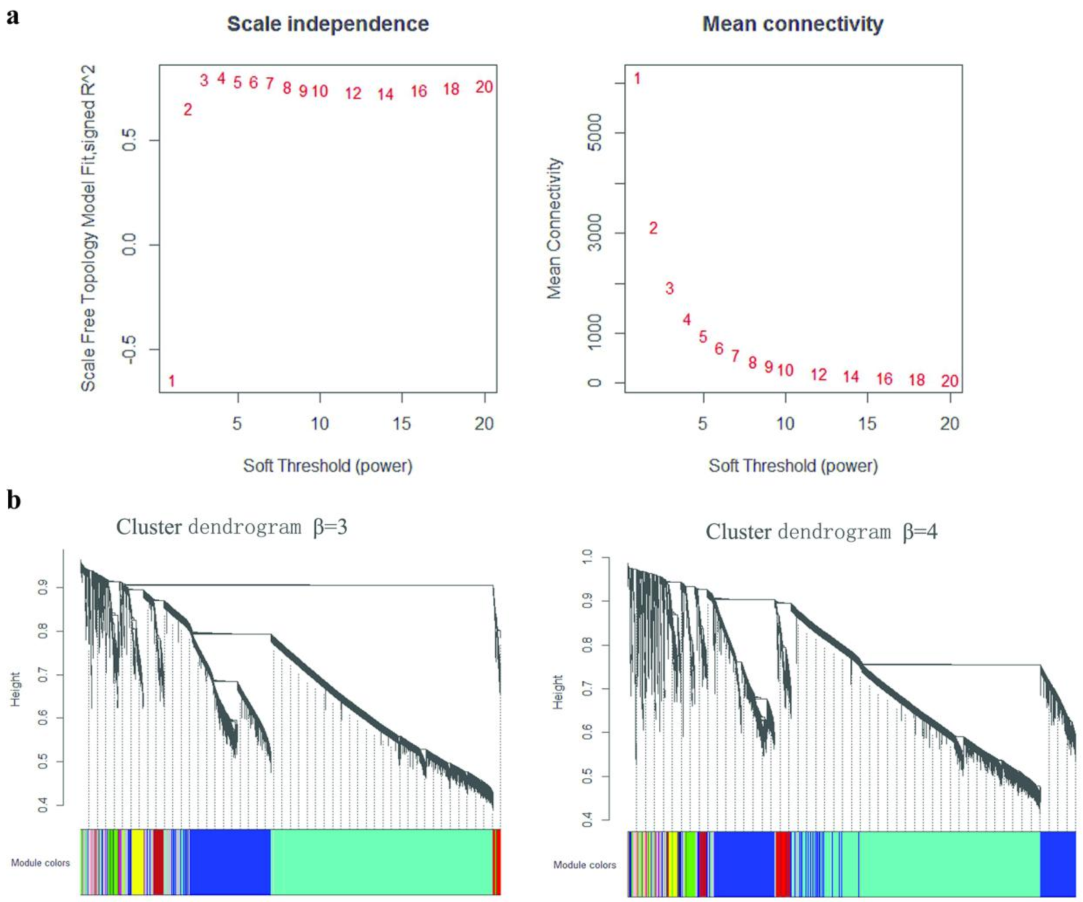

Fig. S1 Robustness assessment of the co-expression module. (**a**) Network topological model for selecting soft-thresholding power. The left panel shows the scale-free indices (y-axis) for different soft-thresholding powers (x-axis). The right panel displays the mean connectivity (degrees, y-axis) for different soft-thresholding powers (x-axis). (**b**) Hierarchical clustering dendrogram and dynamic tree-cut modules using power transformations three and four. This results in 31 and 40 relatively large modules, respectively, compared with using $\beta = 5$.

2. Supplementary Figure 2

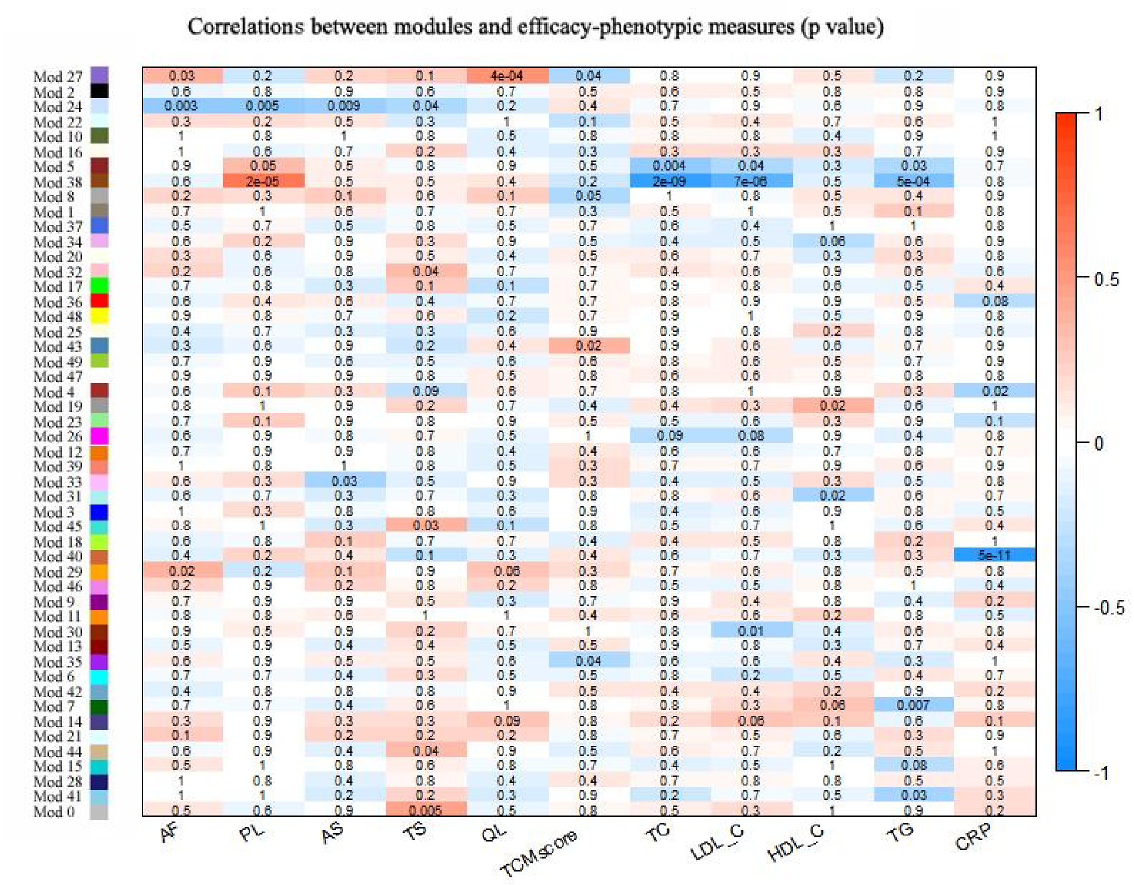

Fig. S2 Heatmap showing the univariate regression model-based correlation p-value between the modules and clinical phenotypes. Each row corresponds to a module eigengene, and each column corresponds to the phenotype. The table was color-coded using a correlation coefficient according to the legend.

3. Supplementary Figure 3

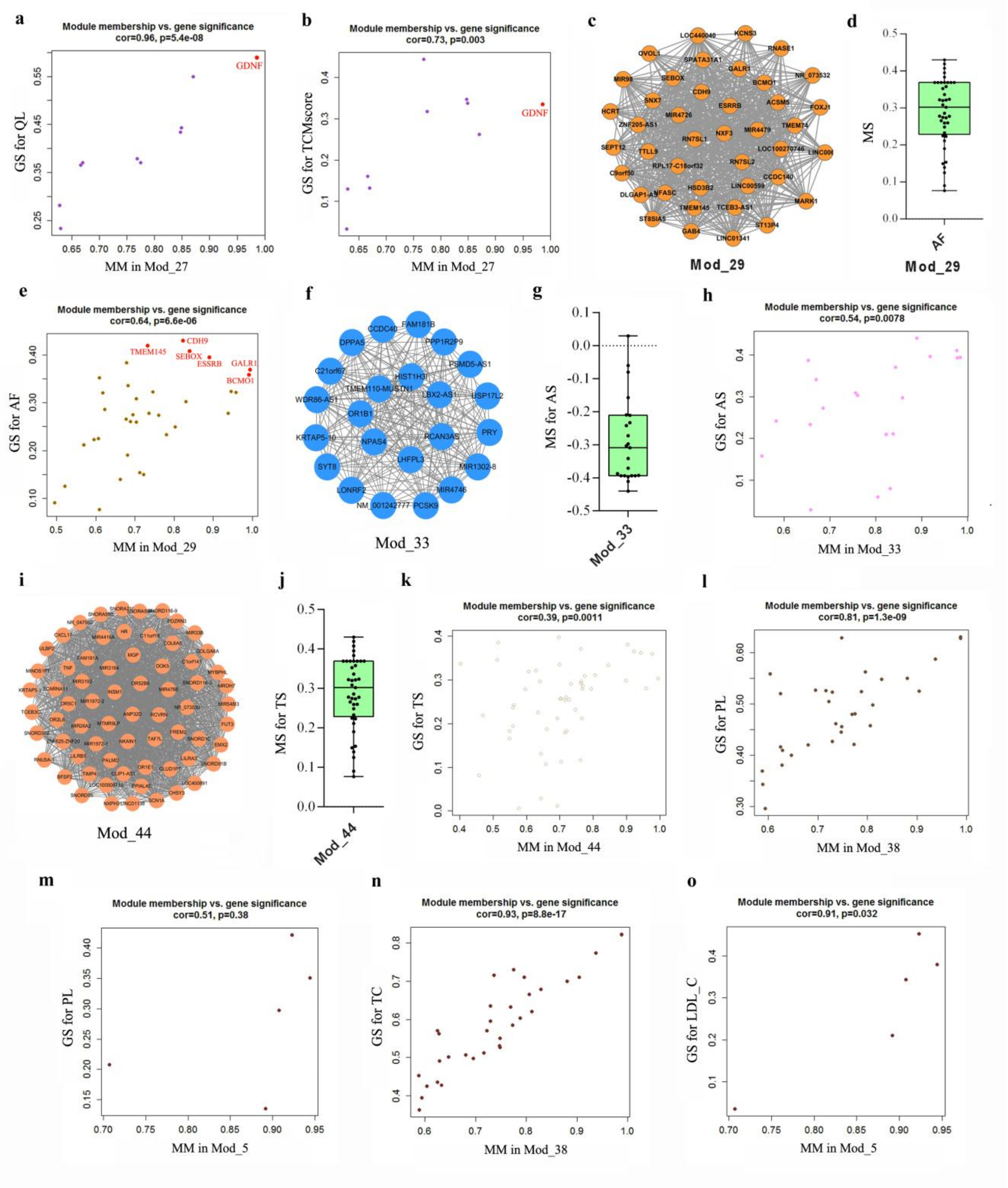

Fig. S3 *Phe-module*s and their correlations with module membership. (**a,b**) Positive correlation between MM in the Angina pectoris module of Mod_27 (x-axis) and gene significance (GS) for QL and TCM scores (y-axis). The red points highlight the GDNF hub genes (MM=0.99). (**c**) Visualization of the AF-specific

Mod_29. (**d**) Positive correlation between the MS of AF-specific Mod_29 and AF. (**e**) Positive correlation between MM in AF-specific Mod_29 (x-axis) and GS in AF (y-axis). The red points indicate the hub genes. (**f-h**) AS-specific Mod_33 and MM/GS correlations. (**i-k**) TS-specific *Mod_44* and its MM/GS correlation. (**l,m**) Positive correlation between MM in serum lipid modules *Mod_38* and *Mod_5* (x-axis) and GS for PL (y-axis). (**n**) Positive correlation between MM in *Mod_38* and GS in the TC (y-axis). (**o**) Positive correlation between MM in *Mod_5* and GS for LDL-C (y-axis).

4. Supplementary Figure 4

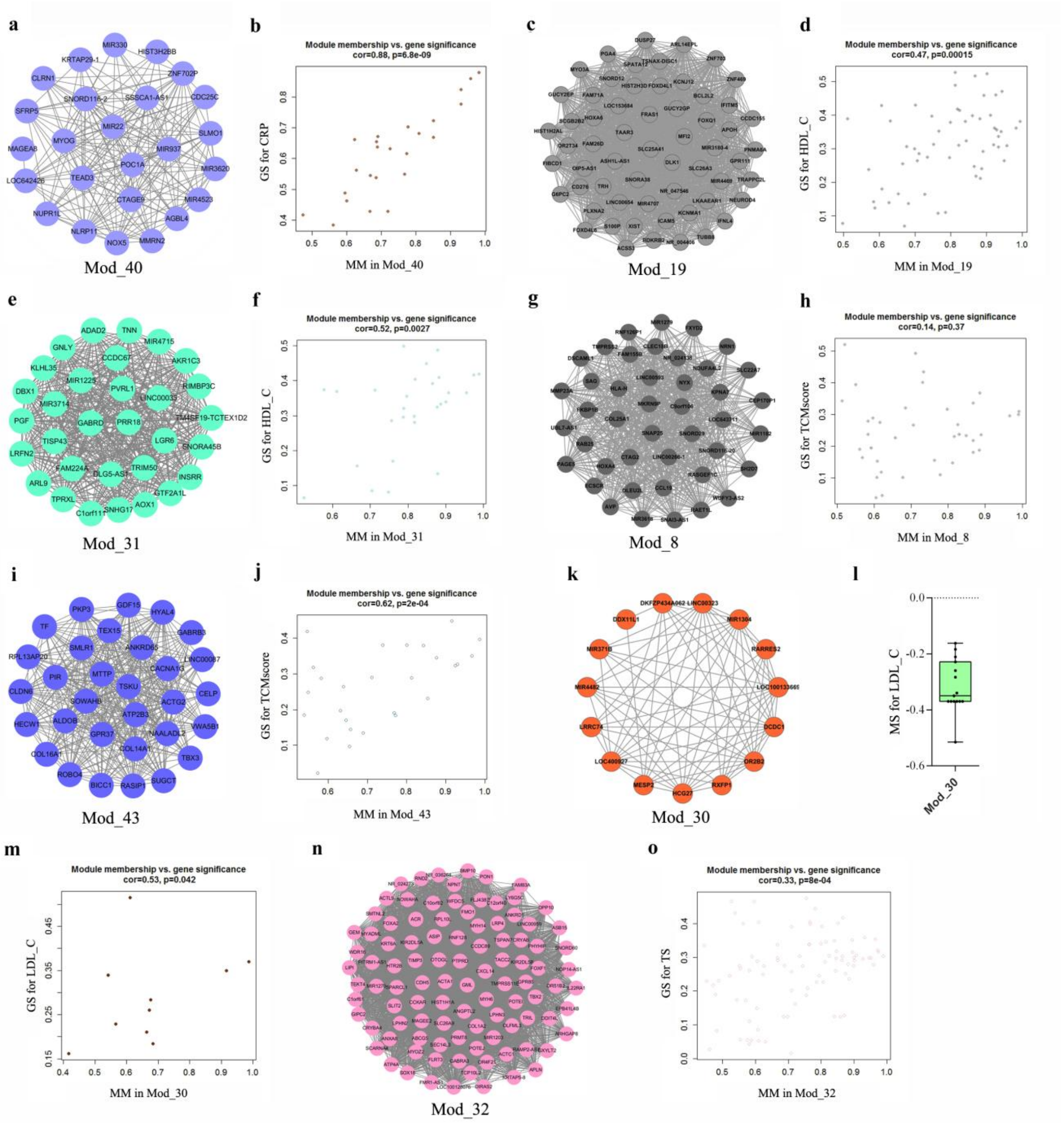

Fig. S4 *Phe-module*s and their correlations with module membership. (**a, b**) CRP module *Mod_40* and its MM/GS correlation. (**c-f**) HDL-C-specific *Mod_19* and *Mod_31* and their MM/GS correlations. (**g-j**) CMM-score-specific *Mod_8* and *Mod_43* and their MM/GS correlations. (**k-m**) LDL-C-specific *Mod_40* and its MM/GS correlation. (**n-o**) TS-specific *Mod_32* and its MM/GS correlation.

5. Supplementary Figure 5

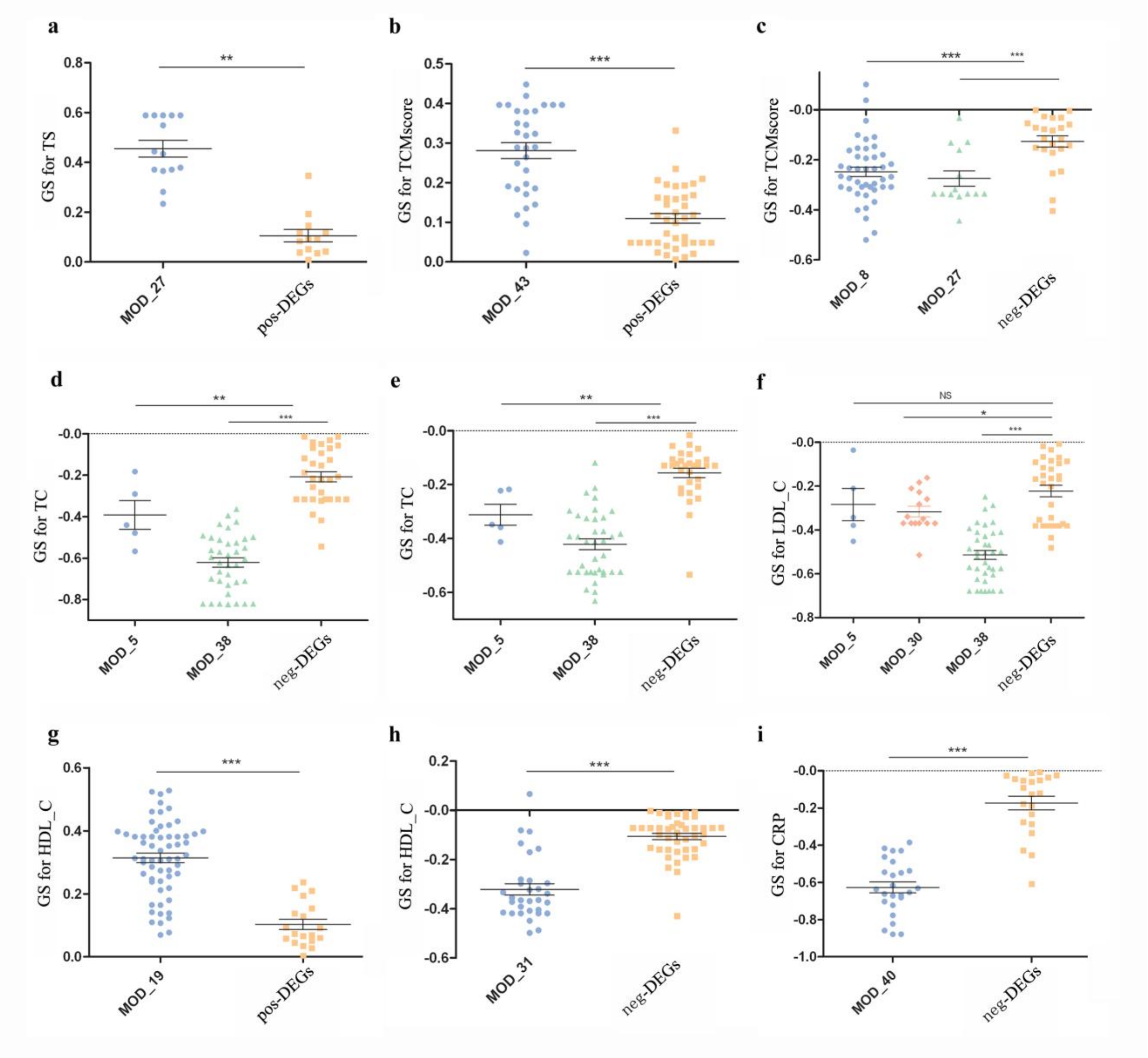

Fig. S5 GS of *Phe-module*s compared with DEGs. For TS, TCM score, TC, LDL_C, HDL, and CRP clinical phenotypes, positively and negatively correlated *Phe-module*s had higher GS than positively or negatively correlated groups of DEGs.

6. Table S3

Table. S3 Correlations between modules and efficacy-phenotypic measures (Pearson Correlation Coefficient)

	AF	PL	AS	TS	QL	TCMscore	TC	LDL_C	HDL_C	TG	CRP
MEbisque4	0.0628	0.0048	0.0838	−0.0644	0.0756	−0.1737	0.1145	−0.0083	0.1069	0.2650	0.0399
MEblack	−0.0841	−0.0488	−0.0164	−0.0998	−0.0595	0.1161	0.0994	0.1097	0.0440	0.0527	0.0159
MEblue	0.0101	0.1653	−0.0513	0.0325	−0.0858	0.0215	−0.1287	−0.0966	−0.0297	0.0426	−0.1060
MEbrown	−0.0784	0.2491	0.1671	−0.2794	0.0934	0.0720	0.0343	−0.0040	0.0272	0.1934	−0.3902
MEbrown4	0.0308	0.3254	0.1128	−0.0439	0.0198	−0.1021	−0.4566	−0.3369	−0.1866	−0.3527	−0.0544
MEcyan	−0.0739	−0.0691	−0.1374	0.1704	−0.1194	−0.1083	−0.0529	−0.2374	−0.1243	0.1520	0.0614
MEdarkgreen	−0.0554	−0.0544	−0.1286	0.0878	0.0063	0.0367	0.0436	0.1837	0.3085	−0.4373	0.0406
MEdarkgrey	0.2201	0.1783	0.2568	0.0967	0.2532	−0.3226	0.0030	−0.0499	0.1215	0.1570	0.0216
MEdarkmagenta	0.0557	−0.0188	−0.0317	0.1219	−0.1603	−0.0753	0.0217	0.1300	0.0354	−0.1420	0.2103
MEdarkolivegreen	0.0099	−0.0392	−0.0091	0.0336	−0.1276	−0.0441	0.0450	0.0450	−0.1439	−0.0197	−0.0040
MEdarkorange	−0.0472	0.0455	0.0870	0.0088	−0.0047	0.1341	0.0814	0.0829	0.2288	0.0327	−0.1236
MEdarkorange2	−0.0624	−0.0212	0.0225	−0.0426	−0.1370	0.1498	−0.0994	−0.0971	−0.0721	0.0351	0.0572
MEdarkred	−0.1131	−0.0165	−0.1304	0.1332	−0.1222	0.1234	−0.0277	−0.0477	−0.1664	0.0718	0.1299
MEdarkslateblue	0.1812	−0.0255	0.1932	0.1630	0.2849	0.0484	0.1971	0.3134	0.2715	−0.0961	0.2484
MEdarkturquoise	0.1264	−0.0054	0.0348	0.0869	−0.0472	−0.0583	−0.1398	−0.1126	−0.0077	−0.2927	0.0818
MEfloralwhite	0.0092	−0.0829	−0.0603	0.2123	−0.1491	−0.1767	0.1773	0.1924	0.1842	−0.0573	0.0149
MEgreen	−0.0557	−0.0451	−0.1697	0.2728	−0.2708	0.0649	0.0126	0.0507	−0.0886	−0.1147	0.1322
MEgreenyellow	−0.0881	−0.0521	0.2501	−0.0701	0.0726	−0.1498	0.1338	0.1261	−0.0375	0.2340	−0.0091
MEgrey60	−0.0394	−0.0039	−0.0305	0.2202	−0.0563	−0.1543	0.1334	0.1647	0.3932	−0.0957	−0.0002
MEivory	0.1842	−0.0981	−0.0299	0.1045	−0.1309	0.1152	0.0976	0.0665	−0.1824	0.1635	−0.0403
MElightcyan	0.2569	−0.0177	0.2281	0.2064	0.2154	−0.0513	0.0740	0.1146	−0.0964	0.1728	0.0220
MElightcyan1	0.1681	0.1965	0.1250	−0.1769	−0.0086	−0.2671	0.1190	0.1285	0.0601	0.0879	−0.0051
MElightgreen	−0.0575	0.2740	−0.0147	0.0329	−0.0137	0.1176	−0.1029	−0.0992	0.1922	0.0112	−0.2461
MElightsteelblue1	−0.4732	−0.4486	−0.4232	−0.3380	−0.1955	0.1494	−0.0626	−0.0270	−0.0782	0.0148	−0.0415
MElightyellow	−0.1426	−0.0658	−0.1595	−0.1682	−0.0847	−0.0269	0.0269	0.0382	0.2277	−0.0530	−0.0996
MEmagenta	−0.0934	−0.0194	−0.0444	0.0756	−0.1190	0.0072	−0.2851	−0.2903	−0.0294	−0.1403	0.0444
MEmediumpurple3	0.3636	−0.2080	0.2169	0.2628	0.5561	−0.3372	−0.0343	0.0144	0.1260	−0.2149	0.0140
MEmidnightblue	−0.0073	−0.0387	−0.1412	0.0506	−0.1283	0.1326	0.0573	0.0472	−0.0334	0.1096	0.1120
MEorange	0.3744	−0.2039	0.2716	−0.0191	0.3138	0.1644	0.0693	0.0845	−0.0532	0.1053	0.0403
MEorangered4	−0.0239	0.1120	−0.0246	0.2333	0.0610	−0.0016	−0.0411	−0.3971	−0.1343	0.0895	0.0449
MEpaleturquoise	−0.0977	−0.0760	−0.1854	−0.0770	−0.1682	0.0384	0.0430	0.0956	−0.3957	0.0795	0.0559
MEpink	0.2006	−0.0831	−0.0443	0.3433	−0.0670	0.0579	0.1341	0.1009	0.0151	0.0982	−0.0832
MEplum1	0.0909	0.1762	−0.3606	−0.1216	−0.0186	0.1894	−0.1491	−0.1244	0.1888	−0.1098	0.0511
MEplum2	0.0780	0.2020	0.0163	0.1935	0.0282	−0.1103	−0.1454	−0.1246	−0.3138	0.1013	0.0172
MEpurple	0.0930	0.0227	0.1093	0.1136	−0.0879	−0.3392	−0.0948	−0.0979	0.1508	−0.1608	0.0040
MEred	−0.0838	0.1449	0.0932	−0.1545	−0.0703	0.0680	0.0499	0.0168	−0.0305	0.1193	−0.2918
MEroyalblue	−0.1090	0.0761	−0.1232	−0.0456	−0.1199	0.0591	−0.0942	−0.1532	0.0089	0.0028	0.0328
MEsaddlebrown	−0.0984	0.6457	0.1064	0.1023	0.1447	−0.2274	−0.8042	−0.6646	−0.1252	−0.5427	−0.0416

MEsalmon	-0.0042	0.0395	-0.0061	-0.0498	-0.1227	0.1656	0.0700	0.0689	-0.0234	0.0966	0.0243
MEsienna3	-0.1412	0.2122	0.1450	-0.2686	-0.1599	0.1524	-0.0918	-0.0587	-0.1657	0.1782	-0.8445
MEskyblue	-0.0100	-0.0082	-0.1965	0.2131	-0.1726	0.0256	-0.2108	-0.0725	-0.1123	-0.3669	0.1841
MEskyblue3	-0.1564	-0.0329	-0.0391	-0.0432	-0.0173	0.1231	0.1326	0.1490	0.2207	0.0264	0.1984
MEsteelblue	-0.1806	-0.0887	-0.0183	-0.2140	0.1487	0.3702	0.0311	0.0811	-0.0919	0.0612	0.0225
MEtan	-0.0778	-0.0141	-0.1324	0.3334	0.0196	-0.1228	0.0820	0.0732	-0.1984	0.1211	-0.0003
MEturquoise	0.0477	0.0103	-0.1673	0.3610	-0.2778	0.0397	-0.1061	-0.0558	-0.0031	-0.1014	0.1320
MEviolet	0.2351	0.0321	0.2270	0.0446	0.2346	0.0522	-0.1153	-0.1172	0.0537	0.0018	-0.1329
MEwhite	0.0125	-0.0122	0.0272	-0.0400	0.1060	0.0409	0.0904	0.0873	0.0458	0.0361	-0.0387
MEyellow	-0.0120	0.0459	-0.0641	0.0957	-0.2357	0.0654	-0.0145	-0.0104	-0.1072	0.0266	-0.0500
MEyellowgreen	-0.0694	-0.0148	-0.0807	-0.1083	-0.0835	0.0927	0.0502	0.0810	0.1046	-0.0557	0.0171

Table. S3 Correlations between modules and efficacy-phenotypic measures (p-value)-continued

	AF	PL	AS	TS	QL	TCMscore	TC	LDL_C	HDL_C	TG	CRP
MEbisque4	0.7118	0.9775	0.6219	0.7048	0.6564	0.3038	0.4998	0.9610	0.5287	0.1129	0.8145
MEblack	0.6209	0.7744	0.9232	0.5566	0.7264	0.4938	0.5582	0.5182	0.7961	0.7569	0.9257
MEblue	0.9525	0.3281	0.7628	0.8485	0.6135	0.8995	0.4479	0.5694	0.8616	0.8023	0.5322
MEbrown	0.6445	0.1370	0.3228	0.0940	0.5824	0.6718	0.8405	0.9812	0.8730	0.2514	0.0169
MEbrown4	0.8563	0.0494	0.5062	0.7962	0.9073	0.5476	0.0045	0.0415	0.2689	0.0323	0.7490
MEcyan	0.6637	0.6845	0.4173	0.3134	0.4814	0.5236	0.7558	0.1571	0.4635	0.3692	0.7181
MEdarkgreen	0.7447	0.7493	0.4480	0.6053	0.9707	0.8294	0.7980	0.2765	0.0632	0.0068	0.8117
MEdarkgrey	0.1906	0.2910	0.1250	0.5691	0.1305	0.0515	0.9859	0.7693	0.4737	0.3533	0.8990
MEdarkmagenta	0.7431	0.9119	0.8523	0.4721	0.3432	0.6577	0.8988	0.4431	0.8352	0.4020	0.2116
MEdarkolivegreen	0.9537	0.8176	0.9573	0.8433	0.4516	0.7956	0.7916	0.7915	0.3954	0.9078	0.9814
MEdarkorange	0.7816	0.7892	0.6088	0.9589	0.9778	0.4287	0.6321	0.6256	0.1731	0.8475	0.4662
MEdarkorange2	0.7138	0.9010	0.8947	0.8021	0.4188	0.3761	0.5584	0.5677	0.6717	0.8365	0.7365
MEdarkred	0.5052	0.9228	0.4419	0.4321	0.4713	0.4670	0.8709	0.7792	0.3249	0.6729	0.4436
MEdarkslateblue	0.2830	0.8809	0.2519	0.3350	0.0875	0.7761	0.2424	0.0589	0.1040	0.5714	0.1383
MEdarkturquoise	0.4561	0.9746	0.8378	0.6092	0.7817	0.7319	0.4094	0.5072	0.9641	0.0787	0.6303
MEfloralwhite	0.9568	0.6257	0.7228	0.2070	0.3784	0.2954	0.2938	0.2538	0.2750	0.7362	0.9303
MEgreen	0.7433	0.7909	0.3153	0.1023	0.1050	0.7028	0.9411	0.7655	0.6019	0.4992	0.4353
MEgreenyellow	0.6041	0.7595	0.1355	0.6799	0.6692	0.3762	0.4299	0.4572	0.8255	0.1634	0.9572
MEgrey60	0.4697	0.6071	0.8707	0.0046	0.5338	0.8061	0.5146	0.3420	0.9724	0.8522	0.2403
MEivory	0.8167	0.9817	0.8579	0.1903	0.7405	0.3618	0.4311	0.3299	0.0161	0.5732	0.9991
MElightcyan	0.2750	0.5636	0.8607	0.5381	0.4400	0.4973	0.5654	0.6956	0.2798	0.3335	0.8128
MElightcyan1	0.1248	0.9170	0.1745	0.2203	0.2003	0.7631	0.6633	0.4994	0.5702	0.3066	0.8971
MElightgreen	0.3199	0.2438	0.4610	0.2950	0.9597	0.1100	0.4830	0.4483	0.7239	0.6049	0.9760
MElightsteelblue1	0.7353	0.1007	0.9312	0.8467	0.9360	0.4881	0.5444	0.5592	0.2543	0.9475	0.1420
MElightyellow	0.0031	0.0054	0.0091	0.0407	0.2462	0.3776	0.7128	0.8737	0.6456	0.9307	0.8072
MEmagenta	0.3997	0.6989	0.3457	0.3196	0.6183	0.8745	0.8745	0.8224	0.1753	0.7555	0.5574
MEmediumpurple3	0.5826	0.9092	0.7943	0.6563	0.4829	0.9661	0.0871	0.0813	0.8628	0.4074	0.7941

MEmidnightblue	0.0270	0.2167	0.1972	0.1161	0.0004	0.0413	0.8402	0.9327	0.4573	0.2016	0.9345
MEorange	0.9660	0.8200	0.4045	0.7660	0.4491	0.4341	0.7362	0.7813	0.8443	0.5186	0.5094
MEorangered4	0.0224	0.2260	0.1039	0.9106	0.0586	0.3310	0.6836	0.6189	0.7546	0.5351	0.8127
MEpaleturquoise	0.8882	0.5094	0.8850	0.1645	0.7201	0.9923	0.8090	0.0150	0.4281	0.5982	0.7920
MEpink	0.5649	0.6546	0.2719	0.6507	0.3197	0.8215	0.8005	0.5734	0.0153	0.6398	0.7423
MEplum1	0.2338	0.6249	0.7948	0.0375	0.6936	0.7333	0.4289	0.5524	0.9292	0.5632	0.6245
MEplum2	0.5927	0.2968	0.0283	0.4735	0.9132	0.2615	0.3786	0.4632	0.2632	0.5176	0.7637
MEpurple	0.6464	0.2305	0.9235	0.2513	0.8686	0.5159	0.3906	0.4624	0.0586	0.5507	0.9194
MEred	0.5842	0.8940	0.5197	0.5033	0.6051	0.0400	0.5767	0.5645	0.3729	0.3416	0.9815
MEroyalblue	0.6219	0.3921	0.5833	0.3613	0.6793	0.6891	0.7695	0.9215	0.8576	0.4820	0.0797
MEsaddlebrown	0.5207	0.6543	0.4675	0.7889	0.4797	0.7282	0.5793	0.3653	0.9581	0.9867	0.8472
MEsalmon	0.5623	0.0000	0.5308	0.5469	0.3929	0.1758	0.0000	0.0000	0.4604	0.0005	0.8070
MEsienna3	0.9804	0.8163	0.9713	0.7698	0.4696	0.3272	0.6806	0.6852	0.8909	0.5694	0.8867
MEskyblue	0.4044	0.2074	0.3917	0.1079	0.3444	0.3679	0.5888	0.7302	0.3271	0.2912	0.0000
MEskyblue3	0.9533	0.9614	0.2436	0.2053	0.3069	0.8807	0.2105	0.6698	0.5080	0.0255	0.2754
MEsteelblue	0.3554	0.8468	0.8185	0.7998	0.9191	0.4681	0.4339	0.3787	0.1893	0.8766	0.2392
MEtan	0.2848	0.6015	0.9143	0.2035	0.3798	0.0241	0.8550	0.6331	0.5886	0.7190	0.8950
MEturquoise	0.6472	0.9338	0.4346	0.0438	0.9082	0.4690	0.6296	0.6669	0.2392	0.4751	0.9984
MEviolet	0.7794	0.9518	0.3224	0.0282	0.0959	0.8156	0.5318	0.7429	0.9856	0.5502	0.4360
MEwhite	0.1613	0.8503	0.1767	0.7932	0.1622	0.7591	0.4967	0.4895	0.7524	0.9915	0.4329
MEyellow	0.9413	0.9427	0.8729	0.8139	0.5322	0.8098	0.5945	0.6074	0.7879	0.8319	0.8203
MEyellowgreen	0.9437	0.7876	0.7061	0.5730	0.1603	0.7006	0.9320	0.9511	0.5276	0.8759	0.7690

Table. S3 Correlations between modules and efficacy-phenotypic measures (ajusted p-value, FDR)-continued

	AF	PL	AS	TS	QL	TCMscore	TC	LDL_C	HDL_C	TG	CRP
MEbisque4	9.9E-01	9.9E-01	9.9E-01	9.9E-01	9.9E-01	9.9E-01	9.9E-01	9.9E-01	9.9E-01	9.9E-01	9.9E-01
MEblack	9.9E-01	9.9E-01	9.9E-01	9.9E-01	9.9E-01	9.9E-01	9.9E-01	9.9E-01	9.9E-01	9.9E-01	9.9E-01
MEblue	9.9E-01	9.9E-01	9.9E-01	9.9E-01	9.9E-01	9.9E-01	9.9E-01	9.9E-01	9.9E-01	9.9E-01	9.9E-01
MEbrown	9.9E-01	9.9E-01	9.9E-01	9.9E-01	9.9E-01	9.9E-01	9.9E-01	9.9E-01	9.9E-01	9.9E-01	5.8E-01
MEbrown4	9.9E-01	9.0E-01	9.9E-01	9.9E-01	9.9E-01	9.9E-01	2.8E-01	8.1E-01	9.9E-01	7.7E-01	9.9E-01
MEcyan	9.9E-01	9.9E-01	9.9E-01	9.9E-01	9.9E-01	9.9E-01	9.9E-01	9.9E-01	9.9E-01	9.9E-01	9.9E-01
MEdarkgreen	9.9E-01	9.9E-01	9.9E-01	9.9E-01	9.9E-01	9.9E-01	9.9E-01	9.9E-01	9.9E-01	3.4E-01	9.9E-01
MEdarkgrey	9.9E-01	9.9E-01	9.9E-01	9.9E-01	9.9E-01	9.1E-01	9.9E-01	9.9E-01	9.9E-01	9.9E-01	9.9E-01
MEdarkmagenta	9.9E-01	9.9E-01	9.9E-01	9.9E-01	9.9E-01	9.9E-01	9.9E-01	9.9E-01	9.9E-01	9.9E-01	9.9E-01
MEdarkolivegreen	9.9E-01	9.9E-01	9.9E-01	9.9E-01	9.9E-01	9.9E-01	9.9E-01	9.9E-01	9.9E-01	9.9E-01	9.9E-01
MEdarkorange	9.9E-01	9.9E-01	9.9E-01	9.9E-01	9.9E-01	9.9E-01	9.9E-01	9.9E-01	9.9E-01	9.9E-01	9.9E-01
MEdarkorange2	9.9E-01	9.9E-01	9.9E-01	9.9E-01	9.9E-01	9.9E-01	9.9E-01	9.9E-01	9.9E-01	9.9E-01	9.9E-01
MEdarkred	9.9E-01	9.9E-01	9.9E-01	9.9E-01	9.9E-01	9.9E-01	9.9E-01	9.9E-01	9.9E-01	9.9E-01	9.9E-01
MEdarkslateblue	9.9E-01	9.9E-01	9.9E-01	9.9E-01	9.9E-01	9.9E-01	9.9E-01	9.5E-01	9.9E-01	9.9E-01	9.9E-01
MEdarkturquoise	9.9E-01	9.9E-01	9.9E-01	9.9E-01	9.9E-01	9.9E-01	9.9E-01	9.9E-01	9.9E-01	9.9E-01	9.9E-01
MEfloralwhite	9.9E-01	9.9E-01	9.9E-01	9.9E-01	9.9E-01	9.9E-01	9.9E-01	9.9E-01	9.9E-01	9.9E-01	9.9E-01

MEgreen	9.9E-01	9.9E-01	9.9E-01	9.9E-01	9.9E-01	9.9E-01	9.9E-01	9.9E-01	9.9E-01	9.9E-01	9.9E-01
MEgreenyellow	9.9E-01	9.9E-01	9.9E-01	9.9E-01	9.9E-01	9.9E-01	9.9E-01	9.9E-01	9.9E-01	9.9E-01	9.9E-01
MEgrey60	9.9E-01	9.9E-01	9.9E-01	2.8E-01	9.9E-01	9.9E-01	9.9E-01	9.9E-01	9.9E-01	9.9E-01	9.9E-01
MEivory	9.9E-01	9.9E-01	9.9E-01	9.9E-01	9.9E-01	9.9E-01	9.9E-01	9.9E-01	5.8E-01	9.9E-01	1.0E+00
MElightcyan	9.9E-01	9.9E-01	9.9E-01	9.9E-01	9.9E-01	9.9E-01	9.9E-01	9.9E-01	9.9E-01	9.9E-01	9.9E-01
MElightcyan1	9.9E-01	9.9E-01	9.9E-01	9.9E-01	9.9E-01	9.9E-01	9.9E-01	9.9E-01	9.9E-01	9.9E-01	9.9E-01
MElightgreen	9.9E-01	9.9E-01	9.9E-01	9.9E-01	9.9E-01	9.9E-01	9.9E-01	9.9E-01	9.9E-01	9.9E-01	9.9E-01
MElightsteelblue1	9.9E-01	9.9E-01	9.9E-01	9.9E-01	9.9E-01	9.9E-01	9.9E-01	9.9E-01	9.9E-01	9.9E-01	9.9E-01
MElightyellow	2.4E-01	2.9E-01	4.2E-01	8.1E-01	9.9E-01	9.9E-01	9.9E-01	9.9E-01	9.9E-01	9.9E-01	9.9E-01
MEmagenta	9.9E-01	9.9E-01	9.9E-01	9.9E-01	9.9E-01	9.9E-01	9.9E-01	9.9E-01	9.9E-01	9.9E-01	9.9E-01
MEmediumpurple3	9.9E-01	9.9E-01	9.9E-01	9.9E-01	9.9E-01	9.9E-01	9.9E-01	9.9E-01	9.9E-01	9.9E-01	9.9E-01
MEmidnightblue	7.1E-01	9.9E-01	9.9E-01	9.9E-01	3.9E-02	8.1E-01	9.9E-01	9.9E-01	9.9E-01	9.9E-01	9.9E-01
MEorange	9.9E-01	9.9E-01	9.9E-01	9.9E-01	9.9E-01	9.9E-01	9.9E-01	9.9E-01	9.9E-01	9.9E-01	9.9E-01
MEorangered4	7.1E-01	9.9E-01	9.9E-01	9.9E-01	9.5E-01	9.9E-01	9.9E-01	9.9E-01	9.9E-01	9.9E-01	9.9E-01
MEpaleturquoise	9.9E-01	9.9E-01	9.9E-01	9.9E-01	9.9E-01	1.0E+00	9.9E-01	5.8E-01	9.9E-01	9.9E-01	9.9E-01
MEpink	9.9E-01	9.9E-01	9.9E-01	9.9E-01	9.9E-01	9.9E-01	9.9E-01	9.9E-01	5.8E-01	9.9E-01	9.9E-01
MEplum1	9.9E-01	9.9E-01	9.9E-01	8.1E-01	9.9E-01	9.9E-01	9.9E-01	9.9E-01	9.9E-01	9.9E-01	9.9E-01
MEplum2	9.9E-01	9.9E-01	7.1E-01	9.9E-01	9.9E-01	9.9E-01	9.9E-01	9.9E-01	9.9E-01	9.9E-01	9.9E-01
MEpurple	9.9E-01	9.9E-01	9.9E-01	9.9E-01	9.9E-01	9.9E-01	9.9E-01	9.9E-01	9.5E-01	9.9E-01	9.9E-01
MEred	9.9E-01	9.9E-01	9.9E-01	9.9E-01	9.9E-01	8.1E-01	9.9E-01	9.9E-01	9.9E-01	9.9E-01	9.9E-01
MEroyalblue	9.9E-01	9.9E-01	9.9E-01	9.9E-01	9.9E-01	9.9E-01	9.9E-01	9.9E-01	9.9E-01	9.9E-01	9.9E-01
MEsaddlebrown	9.9E-01	9.9E-01	9.9E-01	9.9E-01	9.9E-01	9.9E-01	9.9E-01	9.9E-01	9.9E-01	9.9E-01	9.9E-01
MEsalmon	9.9E-01	2.2E-03	9.9E-01	9.9E-01	9.9E-01	9.9E-01	5.6E-07	1.3E-03	9.9E-01	4.8E-02	9.9E-01
MEsienna3	9.9E-01	9.9E-01	9.9E-01	9.9E-01	9.9E-01	9.9E-01	9.9E-01	9.9E-01	9.9E-01	9.9E-01	9.9E-01
MEskyblue	9.9E-01	9.9E-01	9.9E-01	9.9E-01	9.9E-01	9.9E-01	9.9E-01	9.9E-01	9.9E-01	9.9E-01	2.8E-08
MEskyblue3	9.9E-01	9.9E-01	9.9E-01	9.9E-01	9.9E-01	9.9E-01	9.9E-01	9.9E-01	9.9E-01	7.1E-01	9.9E-01
MEsteelblue	9.9E-01	9.9E-01	9.9E-01	9.9E-01	9.9E-01	9.9E-01	9.9E-01	9.9E-01	9.9E-01	9.9E-01	9.9E-01
MEtan	9.9E-01	9.9E-01	9.9E-01	9.9E-01	9.9E-01	7.1E-01	9.9E-01	9.9E-01	9.9E-01	9.9E-01	9.9E-01
MEturquoise	9.9E-01	9.9E-01	9.9E-01	8.3E-01	9.9E-01	9.9E-01	9.9E-01	9.9E-01	9.9E-01	9.9E-01	1.0E+00
MEviolet	9.9E-01	9.9E-01	9.9E-01	7.1E-01	9.9E-01	9.9E-01	9.9E-01	9.9E-01	9.9E-01	9.9E-01	9.9E-01
MEwhite	9.9E-01	9.9E-01	9.9E-01	9.9E-01	9.9E-01	9.9E-01	9.9E-01	9.9E-01	9.9E-01	1.0E+00	9.9E-01
MEyellow	9.9E-01	9.9E-01	9.9E-01	9.9E-01	9.9E-01	9.9E-01	9.9E-01	9.9E-01	9.9E-01	9.9E-01	9.9E-01
MEyellowgreen	9.9E-01	9.9E-01	9.9E-01	9.9E-01	9.9E-01	9.9E-01	9.9E-01	9.9E-01	9.9E-01	9.9E-01	9.9E-01

7. Table S7

Table S7. Clinical baseline characteristics of patients for RNA sequencing.

Characteristics	Total (N=62)	DHI group (N=41)	Control group (N= 21)	P value
Sex —No. (%)				0.6150
Male	41	28	13	
Female	21	13	8	
Age (yr) (Mean(Std))	58(6)	58(6)	57(7)	0.6779
BMI(kg/m^2) (Mean(Std))	26.05(2.71)	25.86(2.59)	26.41(2.96)	0.4525
Prior intensive combination medical therapy for angina—No. (%)				0.1135
Yes	46	33	13	
No	16	8	8	
Diabetes—No. (%)	24	16	8	0.9433
Hypertension—No. (%)	46	30	16	0.7970
Hyperlipidemia—No. (%)	26	18	8	0.6610
SAQ domains (Mean(Std))				
Angina frequency	64(18)	63(19)	67(15)	0.3494
Physical limitation	71(9)	72(10)	69(7)	0.3370
Angina stability	42(22)	41(24)	43(18)	0.7391
Treatment satisfaction	66(12)	66(13)	65(10)	0.7536
Quality of life	37(17)	36(17)	37(19)	0.8458
TCM syndrome score (Mean(Std))	16.77(4.52)	17.20(4.42)	15.95(4.70)	0.3092
CCS angina class—No. (%)				0.8667
II	54	35	19	
III	8	6	2	
Plasma lipid level (mmol/L)(Mean(Std))				
Total cholesterol	3.98(1.12)	4.08(1.25)	3.78(0.80)	0.3299
LDL-cholesterol	2.41(0.90)	2.50(0.95)	2.23(0.78)	0.2540
HDL-cholesterol	1.21(0.36)	1.21(0.31)	1.20(0.45)	0.8704
Triglyceride	1.56(0.93)	1.68(1.04)	1.33(0.62)	0.1568
hs-C-reactive protein (mg/L)	2.07(3.62)	2.10(4.04)	2.00(2.69)	0.9150
Platelet aggregation rate#	38.43(28.94)	41.44(28.41)	33.00(30.61)	0.4826

The 34 patients from Xuanwu Hospital did not receive the test of platelet aggregation rate.

8. Table S10

Table.S10 The primers of BCMO1 and GALR1 used for qRT-PCR analysis

BCO1-F′	ATGGCAACCGCATACATCCG
BCO1-R′	TCAAACACGATGCAGCCGTC
GALR1-F′	CCACTACTTCTTCACCGTGTCCATG
GALR1-R′	CATAGCAGAAGCAGATGAGCAGGAG

β-actin-F′	TGCGTGACATTAAGGAGAAGCTGTG
β-actin-R′	AGTTGAAGGTAGTTTCGTGGATGCC

9. Legends for supplementary tables

Table S1 The modules of the DHI treatment co-expression network

Table S2 The detailed $Z_{summary}$ values of all modules

Table S3 Correlations between modules and efficacy-phenotypic measures

Table S4 The detailed MM values of *Phe-modules*

Table S5 The upregulated and downregulated DEGs between the DHD30 and DHD0 groups

Table S6 The significantly enriched GO terms and KEGG pathways for *On-modules* and *Phe-modules*

Table S7 Clinical baseline characteristics of patients for RNA sequencing.

Table S8 The gene expression datasets for DHD0, DHD30 and CGD30 groups

Table S9 The clinical phenotypes and their variations after DHI treatment

Table S10 The primers of BCMO1 and GALR1 used for qRT-PCR analysis
